# Zwitterionic 3D-Printed Non-Immunogenic Stealth Microrobots

**DOI:** 10.1002/adma.202003013

**Published:** 2020-08-30

**Authors:** Pol Cabanach, Abdon Pena-Francesch, Devin Sheehan, Ugur Bozuyuk, Oncay Yasa, Salvador Borros, Metin Sitti

**Affiliations:** Physical Intelligence Department Max Planck Institute for Intelligent Systems Stuttgart 70569, Germany; Grup d‘Enginyeria de Materials Institut Químic de Sarrià Universitat Ramon Llull Barcelona 08017, Spain; Physical Intelligence Department Max Planck Institute for Intelligent Systems Stuttgart 70569, Germany; Department of Materials Science and Engineering Robotics Institute University of Michigan Ann Arbor, MI 48109, USA; Physical Intelligence Department Max Planck Institute for Intelligent Systems Stuttgart 70569, Germany; Physical Intelligence Department Max Planck Institute for Intelligent Systems Stuttgart 70569, Germany; Physical Intelligence Department Max Planck Institute for Intelligent Systems Stuttgart 70569, Germany; Grup d‘Enginyeria de Materials Institut Químic de Sarrià Universitat Ramon Llull Barcelona 08017, Spain; Physical Intelligence Department Max Planck Institute for Intelligent Systems Stuttgart 70569, Germany; School of Medicine and School of Engineering Koç University Istanbul 34450, Turkey; Institute for Biomedical Engineering ETH Zurich Zurich 8092, Switzerland

**Keywords:** macrophages, non-immunogenic properties, stealth microrobots, two-photon polymerization, zwitterionic materials

## Abstract

Microrobots offer transformative solutions for non-invasive medical interventions due to their small size and untethered operation inside the human body. However, they must face the immune system as a natural protection mechanism against foreign threats. Here, non-immunogenic stealth zwitterionic microrobots that avoid recognition from immune cells are introduced. Fully zwitterionic photoresists are developed for two-photon polymerization 3D microprinting of hydrogel microrobots with ample functionalization: tunable mechanical properties, anti-biofouling and non-immunogenic properties, functionalization for magnetic actuation, encapsulation of biomolecules, and surface functionalization for drug delivery. Stealth microrobots avoid detection by macrophage cells of the innate immune system after exhaustive inspection (>90 hours), which has not been achieved in any microrobotic platform to date. These versatile zwitterionic materials eliminate a major roadblock in the development of biocompatible microrobots, and will serve as a toolbox of non-immunogenic materials for medical microrobot and other device technologies for bioengineering and biomedical applications.

Untethered (wireless) microscale mobile robots are arising as a promising technology for the biomedical and bioengineering fields.[^1–3^] They have potential to offer transformative solutions for non-invasive, local and active medical diagnosis, therapy and intervention, as their small sizes allow them to navigate inside the deep and hard-to-reach regions of the human body.[^4,5^] A myriad of microscale mobile robots has been developed in recent years, with different actuation strategies, including magnetic,[^6,7^] electric,[^8^] acoustic,[^9,10^] photo-,[^11,12^] thermal,[^13,14^] and chemical[^14–17^] actuation, for diverse medical applications,[^1,2^] such as targeted drug delivery, minimally invasive surgery, and remote sensing. However, many scientific challenges lie ahead before such untethered microrobots are ready for clinical use, such as biocompatibility, biodegradation, navigation in complex biofluids, or penetration of biological barriers.[^18^] Among them, robot interaction with the immune system is a major concern that hurdles their medical operation for long durations. The immune system is prepared to neutralize foreign organisms or materials as a natural protective mechanism, and microrobots are not an exception. When a microrobot enters the body (e.g., bloodstream), it would be rapidly coated with proteins via non-specific adsorption.[^19–22^] Macrophages, a type of immune cells that are on the lookout for pathogens, recognize these protein-coated materials as foreign threats and become activated,[^19^] leading to microrobot phagocytosis[^23–25^] (clearing them and disabling their functions) and eliciting an immune response. Therefore, the activation of macrophages is a major obstacle for developing functional medical microrobots that can operate in vivo for prolonged time.

In order to surpass this first innate defense mechanism, we aim to prevent non-specific protein adsorption on the microrobot surface and avoid their detection by macrophages, as it is the first stage of non-specific immune recognition. Poly(ethylene glycol) (PEG) and its derivatives have been extensively used in drug delivery platforms as the current “gold” standard for low-fouling materials against protein adsorption; however, they ultimately get recognized and cleared by the immune cells due to insufficient anti-fouling properties and due to recently discovered anti-PEG antibodies.[^26,27^] Zwitterionic polymers are emerging as an alternative to current antifouling biomaterials in medical devices,[^28^] implantable materials,[^29^] and drug delivery platforms.[^30^] These polymers, inspired in natural biomolecules and surfaces in protein-rich media (like the cell membrane), consist of monomers with positive and negative charges in their structure (with overall zero net charge), which confer superhydrophilicity, ultralow-fouling, and non-immunogenic properties.[^31^]

Here, we introduce zwitterionic non-immunogenic materials for microrobots that avoid detection and phagocytosis from macrophages. We have developed zwitterionic photoresists for 3D printing of hydrogel microrobots using two-photon polymerization for the first time. The zwitterionic materials presented here allow for ample functionalization: tunable mechanical properties, incorporation of magnetic nanoparticles, encapsulation of biomolecules, and surface functionalization. Furthermore, we demonstrate the stealth properties of our zwitterionic microrobots by analyzing their interactions with macrophages in vitro. The stealth microrobots remain undetected after exhaustive inspection by macrophages (at least 90 hours), which has not been observed in any microrobot platform before.[^23,24,32^] These versatile zwitterionic materials would eliminate a major barrier in the development of biocompatible medical microrobots, and will serve as a toolbox of non-immunogenic materials for the design and fabrication of microrobots and microdevices for bioengineering and medical applications.

We synthesized zwitterionic photoresists based on two types of zwitterions: carboxybetaine (CB) and sulfobetaine (SB). CB is a natural zwitterion containing a quaternary amine and a carboxyl group found in naturally occurring biomolecules, such as amino acids and glycine betaine, which is an osmolyte found in plants.[^33^] SB is a non-natural zwitterion containing a quaternary amine and a sulfonate group, with high pH stability and thermoresponsive behavior.[^34^] Both chemistries have shown low non-specific protein adsorption from blood serum and plasma,[^31^] and therefore they are very attractive to design our zwitterionic photoresists. Inspired by previous work,[^29,31,35^] we synthesized CB and SB methacrylate monomers (Figure 1a), as well as CB and SB dimethacrylate crosslinkers (called CBX and SBX, respectively, Figure 1b), by using monofunctional and difunctional tertiary amines and modifying them to obtain the quaternary amine with the corresponding anion. The synthesis products were purified, and their chemical structures were confirmed by ^1^H NMR spectroscopy (Figures S1-S6, Supporting Information).

Having both zwitterionic monomers (CB and SB) and zwitterionic crosslinkers (CBX and SBX) allowed us to formulate hydrogel photoresists with varying crosslinking ratios without losing the zwitterionic properties (i.e., all-zwitterionic hydrogels), which gave us a broad design space for tuning the mechanical properties and photopolymerization kinetics of our materials. We analyzed CB-based and SB-based photoresists with crosslinking ratios from 5% to 100% (CBX and SBX, respectively) by photorheological characterization (Figure S7, Supporting Information). Due to the superhydrophilic nature of the zwitterionic monomers and crosslinkers and their high solubility in water, we achieved high concentrations (up to 70% w/w) in our water-based zwitterionic photoresists that are not possible in other water-based photoresists due to their low solubility limits. By adjusting the crosslinking ratio and concentration, we can tailor the elastic moduli of CB- and SB-based hydrogels from soft (0.1 kPa) to hard (10 MPa) (Figure 1c), matching the moduli of a wide range of biological tissues. We also observed faster polymerization with increasing crosslinking ratio and overall concentration, which is expected due to a higher concentration of methacrylate reactive groups (Figure 1d). We note that these data are in situ shear measurements during photopolyinerization, and might slightly differ from other reports on crosslinked hydrogel moduli measured a posteriori by other methods.[^35^]

3D microprinting via two-photon polymerization (2PP) is an emerging nanofabrication technique that enables 3D complex polymeric structures with down to 100 nm resolulion and has found broad applications in fabricating photonic crystals, metamaterials, cell scaffolds, microfluidic devices, and microrobots.[^6,36,37^] Briefly, a confined nanoscale voxel within a volume of photoresist is illuminated with focused femtosecond laser pulses following a complex computer-aided design (CAD) file, resulting in the photopolymerization of complex 3D structures (Figure 2a)[^38^] Current commercially available photoresists for 2PP lack chemical versatility, and significant research efforts are being made in the development of new functional photoresists[^36,37^] Our zwitterionic photoresists have multiple advantages over the state-of-the-art natural and synthetic materials for 2PP: i) CB, SB, SBX, and CBX are highly soluble in water, and therefore, they do not require organic solvents and can be polymerized with water-soluble photoinitiators, which are less toxic.[^37^] Furthermore, water-only-compatible materials, such as biomolecules or cells, can be integrated into a single printing step. ii) Natural polymers, including gelatin, chitosan, hyaluronic acid, alginate, etc., have limited methacrylation of functional groups (adjusted by reaction time), which restricts the crosslinking density available to such hydrogels. On the other hand, zwitterionic photoresists have a high density of methacrylate groups, which offers tunable control over the hydrogel chemistry and network density, tunable mechanical properties (from soft to hard), and enhanced printing resolution (hydrogels are more stable and preserve their shape, allowing for smaller structural features). iii) Synthetic hydrogels typically use PEG-based or bisacrylamide-based crosslinkers that have limited solubility in water, and therefore restrict the crosslinking density and the hydrogel mechanical properties. In contrast, CBX and SBX crosslinkers have high solubility in water, which allows for high crosslinking ratios and fabrication of stiffer hydrogels without compromising the zwitterionic properties. iv) CB and CBX are directly functionalizable through their carboxylic groups which, together with their v) non-fouling properties, make them a superior alternative to natural and synthetic materials for bioengineering applications, such as drug functionalization and specific targeting.

We optimized the 3D printing for a range of concentrations and crosslinking ratios to achieve high-resolution microscale features (as examples, see the microprinted Max Planck Institute Minerva symbol and a helical microrobot in Figure 2b, Figure S8, Movie SI, Supporting Information). High printing resolution and full structural reproducibility from CAD files were achieved at higher concentrations and crosslinking ratios due to an increased concentration of reactive species (methacrylate groups) and faster polymerization kinetics, while less concentrated and crosslinked photoresists exhibited lower resolution and structural stability. The 3D-printing was optimized for our current photoresists, but it can be further modified by incorporating high molecular weight zwitterionic prepolymers, thickeners, fillers, other photoinitiators, and different laser parameters (power, scan speed, etc.). However, since this is the first time that zwitterionic polymers have been printed by 2PP, this work focused on essential formulations (consisting purely of CB, CBX, SB, and SBX monomers plus photoinitiator) as a guide for future zwitterionic-based photoresist development.

To evaluate the biocompatibility, antifouling, and non-immunogenic (stealth) properties of the zwitterionic microrobots, we fabricated microrobots from different photoresists: C30 and C100 (carboxybetaine-based with 30% and 100% crosslinking), S30 and S100 (sulfobetaine-based with 30% and 100% crosslinking), and PEG (control group). First, we evaluated the biocompatibility of our custom photoresists through cell viability assays. Viability assays and live/dead staining on the macrophages cultured with our zwitterionic polymers showed no toxicity or anomalous cell behavior (Figure S9, Supporting Information), which confirms that the materials are biocompatible and non-toxic. Next, we evaluated the antifouling properties via cell adhesion and protein adsorption experiments. Zwitterionic materials exhibited low cell adhesion compared to other biocompatible photoresists and surfaces (Figure 3a,b and Figure S10, Supporting Information). After culturing for 48 h, the substrates were gently rinsed and the cells on zwitterionic surfaces were washed away, demonstrating low cell adhesion on our photoresists. We further evaluated the antifouling properties against non-specific protein adsorption: since serum albumin is the major protein in blood,[^39^] we used fluorescent-labeled bovine serum albumin (BSA) and fluorescence microscopy to detect protein adsorption on 3D-printed microrobots (Figure Sll, Supporting Information). While the control microrobots showed fluorescence marking the adsorption of fluorescent BSA proteins (Figure 3c), zwitterionic microrobots showed non-detectable protein adsorption (Figure 3d) demonstrating effective anti-biofouling properties and potential for avoiding immune recognition.[^31^

To evaluate the stealth and non-immunogenic properties, we cultured immune cells with microrobots and monitored the cell-robot interactions, leading to inspection, detection, and capture (phagocytosis), or to inspection, non-detection (stealth), and release (Figure 4a). The immune system is extremely complex and is composed of different cell types performing different functions. We have prioritized interactions with macrophages since they are the first trigger in non-specific innate immune response against synthetic foreign materials (Note SI, Supporting Information), such as implanted devices, synthetic nanoparticles, and microrobots[^25^’^29^’^40^] PEG-based microrobots (current anti-biofouling benchmark) were immediately recognized and phagocyted as soon as the macrophages came in contact with them (Figure 4b and Movie S2, Supporting Information). In contrast, zwitterionic stealth microrobots were not phagocyted after exhaustive inspection (cells probe, manipulate, and move the robots) and they were released back (Figure 4c and Movie S2, Supporting Information). We analyzed each interaction between cells and microrobots and measured a high phagocytosis per cell–robot interaction (≈100%) for control (PEG) microrobots, while zwitterionic stealth microrobots have extremely low phagocytosis per cell-robot interaction (<2%) (Figure 4d). C100 presented some low phagocytosis rate (≈20%, which might be caused by batch synthesis impurities), but despite this, it still outperformed current state-of-the-art photoresist materials for biocompatibility and low fouling (PEG). For a critical evaluation of immunogenicity, these experiments were performed on free-floating robots for several reasons: i) cells can freely probe and move the robots for intensive inspection, ii) free-floating robots are most vulnerable to capture by immune cells (as opposed to constrained robots, which can block phagocytosis due to adhesion to the substrate), and iii) non-actuated robots (for example, static robots that have reached their target destination) represent the most vulnerable scenario for capture (as opposed to fast-swimming robots, which can be faster than migrating macrophages). We further investigated the non-immunogenic properties by analyzing the morphology of macrophages interacting with non-stealth PEG microrobots and stealth zwitterionic microrobots at the early stages of inspection (Figure 4e,f). Macrophages inspecting PEG microrobots exhibited a more aggressive morphology, suggested by distinct surface features (extension of filopodia toward the microrobot and presentation of dorsal ruffles on the cell surface, typically observed in activated macrophages, Figure S12, Supporting Information).[^41^] In contrast, macrophages inspecting zwitterionic microrobots present smoother cell surfaces with few or no filopodia trying to engulf the robot.

These results suggest that macrophages are not activated or aggressive toward zwitterionic microrobots, as they are not recognized as a foreign threat. To provide a more comprehensive analysis of the stealth properties and to better evaluate the non-immunogenicity of our microrobots, we have expanded our study to different immune cells (Movie S2, Supporting Information). We validated the stealth properties of large microrobot arrays against macrophages (Figure 4g and Figure S13, Supporting Information), monocytes (Figure 4h and Figure S14, Supporting Information), and splenocytes, which consist of a diverse collection of immune cells present in the spleen, including T-lymphocytes, B-lymphocytes, dendritic cells, and macrophages (Figure 4i and Figure S15, Supporting Information). After exhaustive inspection, robots were probed and moved around by cells, but we did not observe phagocytosis: stealth robots were released and remained free after inspection. The stealth behavior was consistent after numerous cell-robot interactions (even with multiple cells repeatedly inspecting the same robot) for prolonged times (up to 90 hours). Therefore, zwitterionic photoresists offer a versatile platform for microfabrication to overcome limitations in microrobot design without compromising stealth functionalities. We observed that C30, S30, and S100 microrobots presented similar stealth behavior, indicating that both CB-based and SB-based photoresists are effective against macrophage recognition even with different mechanical properties (low and high crosslinking ratios). Overall, these results highlight the biocompatibility, anti-biofouling, and stealth properties of our custom zwitterionic photoresists, which outperform state-of-the-art materials for microrobot fabrication including commercially available photoresists, current camouflage coating strategies (Figure S16, Supporting Information), and PEG-based materials,[^37^] which are considered the current anti-biofouling and stealth “gold” standard.

In addition to their biocompatibility, anti-biofouling, and non-immunogenic properties, zwitterionic hydrogels can be easily functionalized not only in post-printing processes but also in a single printing step. Here, we demonstrate different functionalities of zwitterionic microrobots integrated by the incorporation of inorganic magnetic nanoparticles, encapsulation of biomolecules, and surface modification.

To remotely actuate and control the microrobots, we incorporated biocompatible superparamagnetic iron oxide nanoparticles (SPIONs) to our photoresist formulation for 3D-microprinting of magnetic zwitterionic nanocomposites in a single step (Figure 5a). We 3D-microprinted our zwitterionic microrobots with a helical shape design and used microscale actuation via magnetic torque (more efficient than magnetic gradient pulling at the microscale)[^42^] which is one of the most common strategies for swimming at the low Reynolds number regime in synthetic microrobots.[^6,43,44^] We used external rotating magnetic fields (10 mT) at specific frequencies (ω) to induce spinning torque on the microrobots and propel them through an aqueous solution (Figure 5b and Movie S3, Supporting Information). At lower frequencies (ω< 10 Hz), we observed rolling-type locomotion increasing linearly with actuation frequency and at a drift angle θ≈ 45° (between magnetic actuation axis and microrobot locomotion axis) caused by friction with the substrate (wall effect) (Figure 5c). At the optimum frequency range 10 <ω <13 Hz, the magnetic torque overcomes the substrate friction and we achieved corkscrew-type locomotion through the fluid with zero drift (Figure 5d). At higher frequencies (ω > 14 Hz, step-out frequency), the microrobot cannot catch up with the actuation frequency and the locomotion was defective (low velocity and moving in random directions). We achieved maximum velocities of 14.3 ±1.1 μm s^−1^ (0.8 ± 0.1 body lengths per second), which is an acceptable swimming performance for a soft magnetic composite microrobot.[^44–46^] Better performance for specific tasks could be achieved by increasing the concentration of magnetic particles in the photoresist, which involves colloidal stability problems, aligning the particles to create anisotropy, or using stronger magnetic materials. SPIONs are biocompatible, but other stronger magnetic nanomaterials typically present toxicity problems.[^47^] However, the current approach already demonstrates the compatibility of our zwitterionic materials with state-of-the-art methods of microscale robot actuation, allowing for locomotion through pre-programmed trajectories (Figure S17, Supporting Information), and provides with a new biocompatible, non-immunogenic material platform for magnetic microrobot designs.

Zwitterionic hydrogels can encapsulate a wide array of biological and bioactive molecules in a single printing step, allowing for fast microrobot functionalization (Figure 5e). By directly adding drugs and proteins to our water-based zwitterionic photoresists, we bypassed protein solubility and stability problems that are common to synthetic photoresists. Other photoresists derived from natural polymers are also compatible with water-soluble biomolecules, but typically have low reactive functional group densities (low methacrylation) that yield highly porous materials that cannot trap the biomolecules (leakage). In contrast, biomolecules remained entrapped in zwitterionic microprinted hydrogels over long periods of time without leakage (Figure S18, Supporting Information) due to a tight polymer network (tunable by zwitterionic crosslink/monomer photoresist formulation). Furthermore, zwitterionic photoresists can encapsulate different biomolecules together, allowing for single-step orthogonal functionalization and simultaneous incorporation of diverse functionalities into the microrobot. As a proof of concept, we incorporated BSA conjugated with different fluorophores as protein models and doxorubicin (DOX) as a cancer drug model altogether into our 3D microstructures in a single printing step. Fluorescence microscopy confirmed the retention of the different entrapped molecules in a single printed microstructure (emission at different wavelengths due to the combined presence of three different fluorophores) (Figure 5f). We note that, unlike other commercially available materials, our printed zwitterionic microstructures did not contain residual fluorescence contamination from photoinitiators and are therefore invisible, avoiding any possible overlap in fluorescence microscopy and allowing functionalization and labeling in a broad range of wavelengths (Figure S19, Supporting Information). This versatile approach for encapsulation of biomolecules makes zwitterionic photoresists attractive for a wide range of biomedical applications for 3D-printed microrobotics, such as targeted drug/gene delivery, imaging, biosensing, enzyme therapy, etc. Future work will explore the development of advanced zwitterionic photoresist formulations to control the release of specific entrapped molecules in microrobots, including biodegradable zwitterionic crosslinkers[^48^] and active polymer networks[^34^]

3D printing of zwitterionic microrobots allows not only for functionalization inside the polymer network but also for surface functionalization of the microstructures. While commercial photoresists typically require multiple steps for surface functionalization, CB and CBX have functionalizable carboxylic acid groups readily available. We use l-ethyl-3-(3-dimethylaininopropyl)carbodiimide and N-hydroxysuccinimide coupling (EDC/NHS) to modify the carboxylic acid groups in CB-based microrobots and conjugate them with different molecules containing a primary amine group (Figure 5g). We demonstrated this concept by conjugating fluorophore probes (Figure S20, Supporting Information) and a model cancer drug DOX through a photocleavable linker[^46^] for on-demand light-triggered drug delivery applications (Figure 5h). Briefly, we bonded a diamine to the carboxylic add group via EDC/NHS coupling, added a photocleavable linker (o-nitrobenzyl group with terminal amine-reactive and alkyne groups), and terminated with azide–modified DOX via click chemistry (azide-alkyne click reaction) (Figure S21, Supporting Information). DOX-functionalized microrobots showed stable fluorescence over time, but upon exposure to 365 nm light, the DOX molecules were released to the media due to the photocleavage of the linker molecules (Figure S22, Supporting Information). This functionalization strategy is particularly useful for on-demand light-triggered drug delivery, however, UV light poses challenges in direct translation into biomedical applications due to limited skin/blood/tissue penetration. Recent advances in optical upconversion of NIR to UV light could be used to enhance penetration depth,[^49^] or the linker could be replaced by other photocleavable molecules that are responsive to different wavelengths. One could argue that extreme functionalization of the microrobot surface might come at the cost of reducing the zwitterionic properties. Since this modification can only be performed in carboxybetaine but not sulfobetaine groups, photoresist formulations with tunable SB/CB composition can be designed to control the surface chemistry and introduce surface modification while preserving a sulfobetaine-dominated zwitterionic surface. The versatile surface functionalization of zwitterionic microrobots can easily be extended to other drugs, antibodies, signaling moieties, or biomolecules for chemical sensing. Moreover, the anti-biofouling properties of zwitterionic surfaces avoid non-specific adsorption of proteins, which improves the efficiency of targeting, signaling, or sensing of the attached biomolecules,[^50^] making zwitterionic microrobots very attractive for targeted therapy and biosensing applications.

Zwitterionic photoresists present new materials solutions for critical biocompatibility and immunogenicity challenges in medical microrobots. Immune clearance by macrophage uptake remains a major roadblock in drug delivery and microrobot-enabled technologies, since it neutralizes nano- and microscale robots, drastically decreasing their operational lifetime and their overall efficiency. New zwitterionic microrobots with stealth properties can remain undetected by macrophages after long and exhaustive inspection (>90 hours), which has not been previously achieved in any microrobot system. By evading macrophage detection, zwitterionic non-immunogenic microrobots can avoid their neutralization by phagocytosis and also avoid triggering a potential immune response. Furthermore, the zwitterionic materials developed here (3D-printed using 2PP for the first time) outperform state-of-the-art and commercially available photoresists, and offer broad tailoring and functionalization for robot locomotion, imaging, and drug release strategies. However, further challenges remain to be addressed, such as faster robots for locomotion in non-Newtonian biofluids, synthesis of dynamic photoresists for controlled degradation, and in vivo demonstrations, which will be part of future work. We envision these zwitterionic photoresists as a new toolbox of non-immunogenic materials for 3D microprinting that will enable a new generation of stealth microrobots, small-scale actuators, and devices for bioengineering and medical applications.

## Experimental Section

### Synthesis of Carboxybetaine Methacrylate (CB)

The synthesis of carboxybetaine methacrylate (CB) was adapted from elsewhere.[^51^]Briefly, 140 mL of acrylic acid (AA) were added to 170 mL of N,N-dimethyl(aminoethyl) methacrylate (DMAEMA) in an ice bath. The solution was stirred for 30 min at 0 °C and 4 h at room temperature. 100 mL of ethanol were added to the reaction and the solution was stirred at room temperature for 12 h. Ethanol was removed at reduced pressure and the resulting liquid was added to a 1 L solution of diethyl ether/trimethylamine (8:2). A white solid precipitated, it was filtered and then cleaned with ethyl ether. The resulting white solid (Figure SI, Supporting Information) was dried under vacuum (85 g, 35% yield). Chemicals were purchased from Sigma Aldrich unless explicitly noted.

### Synthesis of Sulfobetaine Methacrylate (SB)

50 mL of DMAEMA were added to 250 mL of tetrahydrofuran (THF). 44 g of propanesultone were dissolved in 250 mL of THF and then were added to the solution in an ice bath. The solution was stirred overnight. A white solid (Figure S2, Supporting Information) precipitated from the reaction, it was filtered, cleaned with THF, and dried under vacuum (56 g, 73% yield). Chemicals were purchased from Sigma Aldrich unless explicitly noted.

### Synthesis of Carboxybetaine Dimethacrylate (CBX)

The synthesis of carboxybetaine dimethacrylate (CBX) was adapted from a protocol developed in Jiang’s lab[^52^] in which they synthesized a carboxybetaine moiety with two methacrylate groups. First, 11.9 g of N-methyldiethanolamine, 100 mL of toluene, 21.5 g of methacrylic acid and 2 g of hydroquinone were added to a 500 mL reaction flask fitted with a stirrer, condenser, and Dean-Star trap. 14.4 g of methanesulfonic acid were added and the mixture was heated to reflux. After 6 h, the solution was cooled to room temperature. The mixture was neutralized with aqueous sodium hydroxide and the aqueous phase was removed in a decantation funnel. The organic phase was washed with 10% brine solution and it was dried with anhydrous magnesium sulphate. Alumina free base was added to the solution and filtered. Toluene was removed under vacuum to obtain N-methyldiethanolamine dimethacryiate (Figure S3, Supporting Information) as a colorless liquid with a yield of 65% (16.5 g). Next, 16.5 g of N-methyldiethanolamine dimethacrylate were dissolved in 150 mL of acetonitrile in a 500 mL round flask. The solution was purged with nitrogen for 20 min and 15.2 g of t-butyl bromoacetate (TCI Deutschland GmbH) were added to the solution. The solution was purged again with nitrogen during 10 min and the solution was stirred during 48 h at 60 °C. The solvent was removed under vacuum and the resulting liquid was added to 500 mL of diethyl ether, in which a white solid precipitates. This solid (N-methyl-N-di(2-methacryloyloxy-ethyl)-N-l-(t-butyloxycarbonylmethyl), Figure S4, Supporting Information) is cleaned with 250 mL of diethyl ether and dried under vacuum. The yield of this step is 86% (25.0 g). Finally, the tert-butyl protecting group was removed by adding 60 mL of trifluoroacetic acid and 240 mL of dichloromethane to 20 g of *N*-Methyl-*N*-di(2-methacryloyloxy-ethyl)-*N*-l-(t-butyloxycarbonylmethyl). The reaction was stirred for 40 h at room temperature and then the solvent was evaporated under vacuum. 200 mL of acetonitrile were added to the remaining liquid and Amberlite IRN 78 free base was added to the solution to neutralize it. Acetonitrile was removed under vacuum and the resulting liquid was precipitated in diethyl ether to obtain a white solid. This solid (Figure S5, Supporting Information) was cleaned with diethyl ether and was dried in vacuum. The yield of this step was 51% (7.1 g). Chemicals were purchased from Sigma Aldrich unless explicitly noted.

### Synthesis of Sulfobetaine Dimethacrylate (SBX)

The synthesis of SBX was adapted from elsewhere,[^53^] starting from N-methyldiethanolamine dimethacrylate, a mid-product of CBX synthesis. 10 g of N-methyldiethanolamine dimethacrylate were dissolved in 50 mL of anhydrous acetone. 5.75 g of propanesultone were dissolved in 25 mL of anhydrous acetone and were added to the previous solution. The reaction was stirred for 5 h at 60 °C, and a white solid appeared. The solid (Figure S6, Supporting Information) was filtered, cleaned with anhydrous acetone, and dried in vacuum. The yield was 64% (10.0 g). Chemicals were purchased from Sigma Aldrich unless explicitly noted.

### UV Photopolymerization of Zwitterionic Photoresists

Photoresists formulations of CB/CBX and SB/SBX in deionized water with concentrations of 10%, 17%, 40%, and 60% w/w and variable crosslinking ratio were prepared and investigated. Lithium phenyl-2,4,6-trimethylben zoylphosphinate (LAP, Tokyo Chemical Industry Co. Ltd.) photoinitiator was added to the formulation to a 4.3% w/w to monomer content. In situ photopolymerization was analyzed in a TA Instruments Discovery HR-2 rheometer with a photorheology accessory and an external UV light source (Omnicure series 2000 UV lamp, broadband 320-500nm). Photorheology measurements were performed at 0.1% strain and a frequency of 10 rad s^−1^.

### 3D Microprinting (Two-Photon Polymerization)

Helical microrobots (single helix, 20 μm long, 5 μm in diameter, 2 μm in thread diameter, 7 μm in pitch) were designed in Solidworks, and CAD files were prepared for printing using Describe software (Nanoscribe GmbH). Water-based zwitterionic photoresists were prepared at the desired crosslinking ratio and concentration, with 4.3% w/w LAP photoinitiator. For example, C100 at 60% w/w photoresists were prepared by dissolving 30 mg of CBX and 1.35 mg of LAP in 20 μL of deionized water and sonicated for 5 min. The photoresist was then placed on a glass slide and transferred for 3D-printing via two-photon polymerization in a Photonic Professional system (Nanoscribe GmbH) with a 63x oil-immersion objective (NA 1.4). For better printing results, printing parameters were optimized to a laser power of 20 mW and scanning speed of 10^4^ μm s^−1^. The photoresist concentration and crosslinking ratio were optimized for optimal resolution, reproducibility, and microstructure stability. The approximated printing time for a single helical microrobot was 20 s. To print magnetic microrobots, dextran-coated 50 nm iron oxide magnetic nanoparticles (Chemicell GmbH) were added to the photoresist formulation prior to printing at a concentration of 12.5 mg mL ^−1^ and were printed using the same parameters. PEG-based microrobots were printed with poly(ethylene glycol) diacrylate (PEGDA, Mn ≈ 250) containing 3% w/v Irgacure 369 photoinitiator. 27.5 mW laser power ad scanning speed of 1.16 × 10^4^ pm s^−1^ were used for all printings. The overall printing rate was measured as ≈10 s for a single helical microrobot.

### Cell Culture

All cell culture was performed in sterile conditions under or within a biosafety cabinet. The cell culture medium used for each cell line was prepared as Dulbecco’s modified Eagle medium (DMEM) (Gibco) supplemented with 10% HI-FBS (heat-inactivated Fetal Bovine Serum) (Gibco) with 1% Penicillin and Streptomycin (Gibco). All cells were stored under standard cell culture conditions, 5% C0_2_, 80% humidity, and 37 °C.

### J774A.1

Murine macrophage cells (J774A.1) were purchased from ATCC. The cells were characterized by surface markers (CD11b, CD80, CD206), morphology, and ability to phagocyte. The cells were thawed from cryopreservation at passage 3. The cellular passages for the experiments was between passages 5 up to passage 25. J774A cells were allowed to reach ≈80% confluence, observed by microscopy. The cellular removal procedure was performed by rinsing with DPBS without Ca^2+^ and Mg^2+^ for 5 min. After aspiration of DPBS, a fresh addition of medium was added. The cells were then removed from the flask by cell scraper and counted with a hemocytometer. A cell suspension of 5 × 10^4^ cells per mL was created.

### THP-1

Human monocyte non-adherent cell line THP-1 cells were purchased from ATCC. The cell culture medium was also DMEM. The cells were thawed from cryopreservation at passage 2. Due to the non-adherent nature of the THP-1 cells, the passage procedure is centrifugation at 400 × g for 5 min. Then the cells were resuspended to a density of 5 × 10^4^ cells per mL in DMEM. The final volume of media and cells added to microrobots was 1 × 10^6^ cells in 2 mL of DMEM.

### Murine Spleen Isolation/Harvest

Mouse spleen was provided by the Facility for Animal Welfare, Veterinary Service and Laboratory Animal Science at the Eberhard Karls Universität Tübingen. Immediately after sacrificing, the spleen was removed and kept in PBS without Ca^2+^/Mg^2+^ at 4 °C. The time between harvest and isolation step was ≈2 h. The spleen was passed through a 70 μm cell strainer (Corning) containing Roswell Park Memorial Institute (RPMI) 1640 medium in a 50 mL conical tube. This suspension was centrifuged at 800 × g for 5 min. The supernatant was aspirated off and the pellet of cells was resuspended in 1 mL of Ammonium-chloride-potassium lysing buffer (ACK) for the lysis of red blood cells. This suspension was kept at room temperature for 5 min. The cell suspension was then diluted with 9 mL of RPMI 1640 and centrifuged again. The cells were then resuspended with fresh RPMI 1640 and counted. The cell density was calculated and prepared as a 1 million cell suspension in 2 mL of RPMI 1640 which was added to the microrobots.

### Cell Viability

J774A.1 cells were allowed to reach ≈80% confluence. The cellular removal procedure was performed by rinsing with Dulbecco's phosphate-buffered saline (DPBS) without Ca^+^ and Mg^+^ for 5 min. After aspiration of DPBS a fresh addition of medium was added. The cells were then removed from the flask by cell scraper and counted by hemocytometer. A cell suspension of 5 × 10^4^ cells per mL was created. The samples were placed within trans-well inserts of 6.5 mm diameter with 8.0 μm pore size. After mixing, the cell suspension was added to the wells of the plate containing samples at 5 × 10^4^ per well. After attachment, the media was aspirated and replaced with 800 μL of media placed into the bottom of the well, 200 μL of media placed into the top compartment of the trans-well insert already containing the sample. The plates were placed into standard culture conditions and incubated for 24 h of exposure time. Following the 24 h exposure time, the plates were removed and visually observed. The media was collected and stored at 4 °C until analyzed for other markers. A batch of 10% water-soluble tetrazolium salt (WST-8) into cell culture media was prepared. Approximately 200 μL of the WST-8 dilution was placed into the wells containing cells. This was incubated at standard culture conditions for ≈1 h before the media was removed and placed into a 96-well plate. This was measured at 450 nm on a spectrophotometer. After the removal of the WST-8 the wells were rinsed with DPBS and a Live/Dead stain was added to the well. This Live/Dead stain was incubated at room temperature for 20 min before being observed using a fluorescence microscope (Nikon Eclipse Ti-E, Tokyo, Japan). The live cells were imaged at 494/518 nm, while the dead cells were imaged at 528/617 nm. Images were analyzed with Fiji software (Image) version 1.52 g).

### Cell Adhesion

J774A.1 cell suspension of 5 × 10^4^ cells per mL was prepared as described above and added to the 24-well cell culture plates containing samples at 5 × 10^4^ per well. After a 24 h exposure time the plates were removed and observed by microscopy. The initial 24 h images were then taken at 10× magnification at the bottom of the plate and the surface of the samples. A subsequent 24 h were allowed to pass and the samples were immersed in DPBS and moved gently to ensure a full rinse. The samples were then placed into fresh medium. The final images were taken at 48 h post cell culture addition and after DPBS rinsing.

### Protein Adsorption

Microrobot arrays were printed from PEGDA, IP-S (commercial photoresist, Nanoscribe GmbH), S30, C30, S100, and C100 photoresists. Microrobots were immersed in 10 μg mL^−1^ albumin from bovine serum (BSA), Alexa Fluor 647 conjugate in PBS for 2 h, and were then rinsed with Dl water.

### Cell Inspection of Microrobots

Cell suspensions were prepared as described above for each cell type. Cell suspension were added to a petri dish containing 3D-printed microrobots. The samples cultured with J774A.1 were kept at standard culture conditions for 1 h to allow attachment. Then the samples were moved to the incubation chamber of a Nikon Spinning Disk microscope and a time-lapse recording was initiated with images taken every 5 min. The samples cultured with THP-1 or splenocytes were moved to the microscope enclosure at standard culture conditions immediately following seeding. The interaction between microrobots and immune cells was monitored, analyzing every contact interaction and whether the microrobots were detected and phagocyted or not detected and released. Arrays of 7x7 microrobots were analyzed in triplicates (at least 147 microrobots for every material formulation). As a representative figure of merit, the number of phagocyted robots was normalized by the number of contact interactions.

### Scanning Electron Microscopy

Samples with cells were fixed with 2.5% v/v glutaraldehyde in lx PBS for 30 min at 4 °C and then rinsed 3 times with lx PBS. After that, they were dehydrated in a series of increasing aqueous ethanol concentrations (20%, 40%, 60%, 80%, and 100%) for 3 min in each solution. Then, the samples were dried using a CO_2_ critical point dryer (Leica EM CPD300, Leica Microsystems, Wetzlar, Germany) and coated with 10 nm gold using a spin coater (Leica EM ACE600, Leica Microsystems, Wetzlar, Germany). Finally, they were examined with a Zeiss Ultra 550 Gemini scanning electron microscope (Carl Zeiss Inc., Oberkochen, Germany) using an accelerating voltage of 5 keV.

### Magnetic Actuation of Microrobots

Although the magnetic nanoparticles in the photoresist were homogeneously distributed, the microrobots were actuated via magnetic torque due to the anisotropic geometry of the microstructures. Magnetic microrobots were actuated using a custom-built five-electromagnetic coil system (5-coil setup: 4 x-y coils and 1 z coil, each 1.6 cm in diameter and 3.5 cm long) mounted on an inverted microscope that generated variable magnetic fields (5x5x1 mm^3^ of workspace, field range of 0-10 mT, and current range of +/-10 A). Electric currents through the coils were calculated to minimize magnetic field spatial gradients. Rotating 10 mT magnetic fields at variable frequency and orientation were used to induce torque to propel and steer the microrobots along programmable trajectories.

### Biomolecule Encapsulation in 3D-Printed Zwitterionic Microstructures

FITC-labeled BSA (Sigma Aldrich), AlexaFluor 647-labeled BSA (AlexaFluor), and DOX (LifeTein LLC) were simultaneously added to the photoresist formulation to a concentration of 1 mg mL^−1^ each. The photoresists were directly transferred to printing with the previous printing parameters without any additional step. The printed microstructures were thoroughly rinsed to remove non-polymerized photoresist and were imaged in a Nikon Ti-E fluorescence microscope.

### Microrobot Functionalization for Light-Triggered Controlled Drug Release

Microrobots were printed with CB30 (i.e., *70%* CB, 30% CBX) and further functionalized with DOX molecules through a photocleavable linker. First, free amine groups were introduced to the surface of microrobots via EDC/NHS coupling to the carboxybetaine carboxylic groups. Microrobots were incubated for 4 h in 10 × 10^−3^ m of l-ethyl-3-(3-dimethylaminopropyl)carbodiimide and 20 × 10^−3^ m of N-hydroxysuccinimide in a 0.1 m MES buffer (pH = 5.5). Microrobots were then rinsed with PBS and incubated in 0.1 m of butyldiamine in PBS overnight, and were then rinsed with Dl water and dimethyl sulfoxide. Next, microrobots were incubated in a solution containing 2.5 × 10 ^−3^ m of l-(5-methoxy-2-nitro-4-prop-2-ynyloxyphenyl)ethyl N-succinimidyl carbonate (a photocleavable *o*-nitrobenzyl linker, LifeTein LLC, Somerset, NJ, USA) for 3 h, and then rinsed with DMSO. Last, copper(l)-catalyzed azide-alkyne click chemistry was performed to bond an azide-modified DOX (LifeTein LLC) with the alkyne end of the photocleavable linker. A 50 × 10^−3^ m of azide-DOX, 100 × 10^−6^ m CuS0_4_, 5 × 10 ^−3^ m sodium ascorbate, and 500 × 10 ^−6^ m tris(3-hydroxypropyltriazolylmethyl)amine solution was prepared and microrobots were incubated in it during 2 h. Samples were rinsed with Dl water to remove excess DOX. Light-triggered DOX release from the functionalized microrobots was performed using a 365 nm UV external light source (55 mW cm^−2^) source. Drug release from the microswimmers was measured using an Inverted fluorescence microscope (Nikon Eclipse Ti-E, Tokyo, Japan). Chemicals were purchased from Sigma Aldrich unless explicitly noted.

## Supplementary Material

Supplementary material

Supplementary video 1

Supplementary video 2

Supplementary video 3

## Figures and Tables

**Figure 1:**
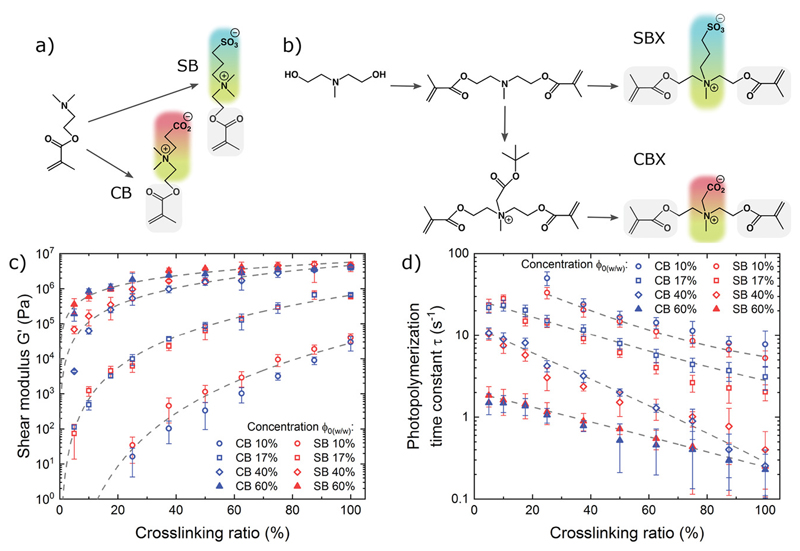
Synthesis and characterization of zwitterionic photoresists. a) Synthesis of sulfobetaine (SB) and carboxybetaine (CB) methacrylate monomers. b) Synthesis of sulfobetaine (SBX) and carboxybetaine (CBX) dimethacrylate crosslinkers, c) Shear modulus and d) photopolymerization kinetics of CB- and SB-based hydrogels as a function of crosslinking ratio and concentration. The error bars represent the standard deviation.

**Figure 2:**
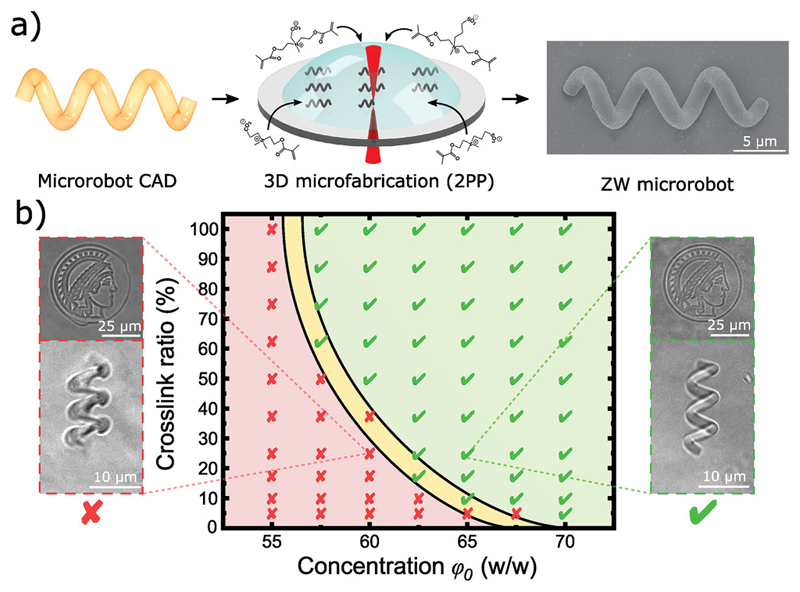
3D microprinting of zwitterionic photoresists using two-photon polymerization. a) Zwitterionic photoresists (containing zwitterionic monomers, zwitterionic crosslinkers, and photoinitiators) were printed into 3D complex microstructures (including helical microrobots) via two-photon polymerization (2PP). b) Printing diagram for optimized resolution as a function of concentration and crosslink ratio. Insets show printed microstructures with sub-optimal resolution (left, red) and optimal resolution for full structural reproducibility (right, green).

**Figure 3:**
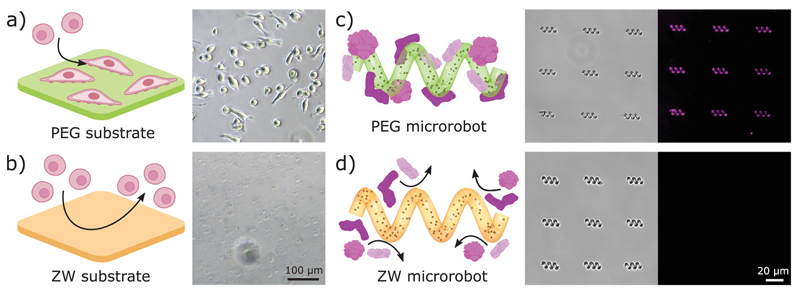
Anti-biofouling microrobots. ab) Cell adhesion on PEG (a) and zwitterionic (ZW) S30 (b) substrates after 48 h culture and rinsing. PEG substrates showed attached cells after rinsing, while cells on ZW substrates were easily washed away. c, d) Protein adsorption on PEG microrobots (c) and zwitterionic (ZW) S30 microrobots (d). Microrobots were immersed in a fluorescent BSA solution for 2 hours and then rinsed. ZW microrobots did not show protein adsorption on their surface (no fluorescent signal).

**Figure 4:**
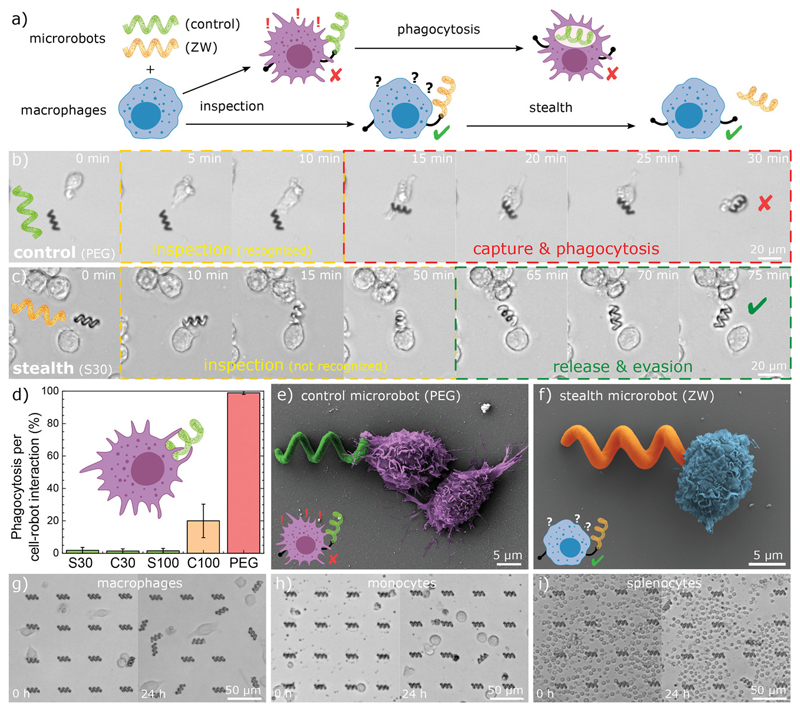
Non-immunogenic stealth microrobots. a) Schematic of cell–robot interaction: control microrobots are captured and phagocytosed by activated macrophages, while undetected stealth microrobots are released after inspection. b) Inspection and capture of control PEC microrobots. After a short inspection time, the microrobot was recognized by the macrophage, captured, and phagocyted. c) Inspection and release of stealth microrobots (S30). After exhaustive inspection, the stealth microrobot was not recognized and it was released by the macrophage, avoiding phagocytosis. d) Phagocytosis rate for different types of microrobots (normalized by cell-robot interactions). The error bars represent standard deviation. e,f) SEM image of macrophage interacting with a non-stealth control robot (e) and with a zwitterionic (S30) stealth microrobot (f) at the early stages of inspection. g) Macrophages, h) monocytes, and i) splenocytes interacting with arrays of zwitterionic (S30) stealth microrobots for 24 hours.

**Figure 5:**
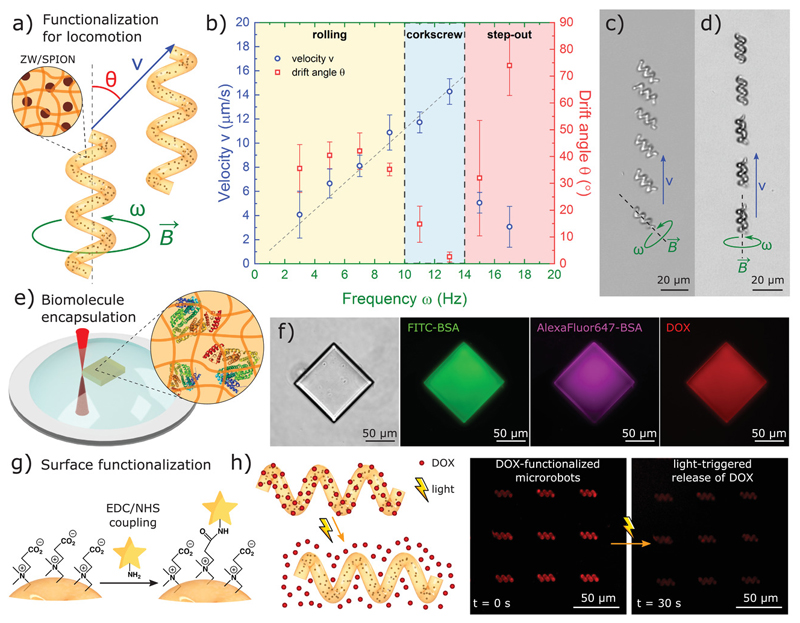
3D-printed multifunctional zwitterionic microrobots. a) Helical microrobots were functionalized with embedded superparamagnetic iron oxide nanoparticles for swimming locomotion via magnetic-torque-based spinning at the low Reynolds number regime. b) Velocity and drift angle as a function of spinning frequency showed rolling, corkscrew, and step-out regimes. c) Rolling locomotion of microrobots (*ω* = 3 Hz). d) Corkscrew locomotion of microrobots (*w* = 13 Hz), e) Encapsulation of biomolecules in a single 3D printing step. f) Three fluorescent biomolecules (FITC-BSA, AlexaFluor 647-BSA, and DOX) simultaneously encapsulated in a 3D-printed microstructure. g) Surface functionalization of microrobots through carboxybetaine functional groups. h) Ultraviolet (UV) light-triggered drug release from DOX functionalized carboxybetaine microrobots, as an example controlled drug release demonstration.
