# Supplementary material for: Zwitterionic 3D-Printed Non-Immunogenic Stealth Microrobots
Source: Adv Mater. Author manuscript; Available in PMC 2021 Mar 29. (PMC7610461; doi:10.1002/adma.202003013)
Supplement: Supplementary material [file EMS104392-supplement-Supplementary_material.pdf]

© 2020 Wiley-VCH GmbH

ADVANCED MATERIALS

Supporting Information

for *Adv. Mater.*, DOI: 10.1002/adma.202003013

Zwitterionic 3D-Printed Non-Immunogenic Stealth Microrobots

Pol Cabanach, Abdon Pena-Francesch, Devin Sheehan, Ugur Bozuyuk, Oncay Yasa, Salvador Borros, and Metin Sitti**

Copyright Wiley-VCH GmbH, 2020.

Supporting Information

Zwitterionic 3D-Printed Non-Immunogenic Stealth Microrobots

Pol Cabanach[#], Abdon Pena-Francesch[#], Devin Sheehan, Ugur Bozuyuk, Oncay Yasa, Salvador Borros[*], Metin Sitti[*]

[#] These authors contributed equally
[*]Correspondence to: sitti@is.mpg.de, salvador.borros@iqs.url.edu

This file includes:

Figures S1 to S22
Note S1

Other Supplementary Materials for this manuscript include the following:

Movies S1 to S3

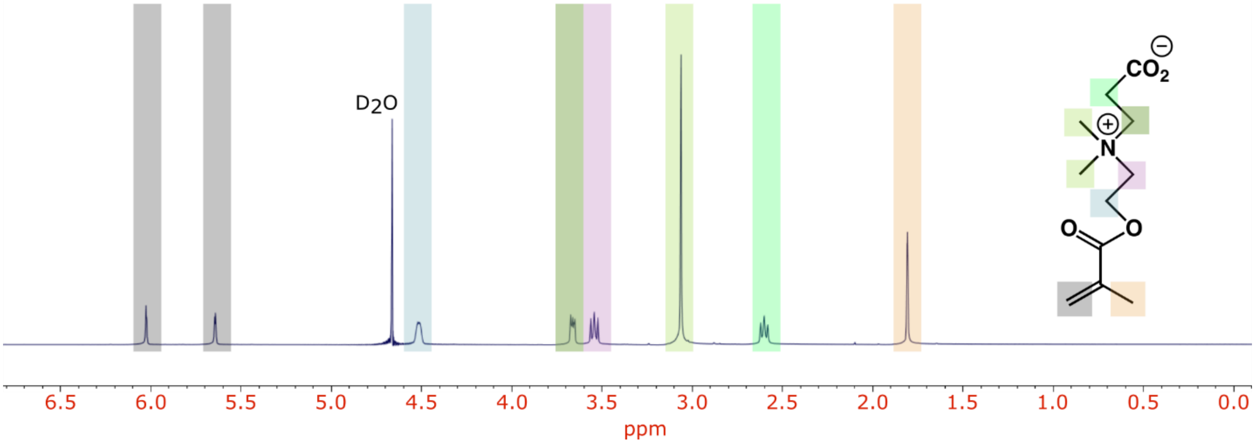

Figure S1: H[1]NMR of carboxybetaine methacrylate (CB) in D_2O.

We confirmed the chemical structure of CB by observing:

- two protons corresponding to the methacrylate double bond (5.6 and 6.1 ppm)
- singlet at 3.05 ppm with an integral 6 that corresponds to the two methyl groups of the quaternary ammonium
- two methyl groups also bonded to the amine appear at 3.6 and 3.7 ppm
- methyl of the methacrylate at 1.7 ppm
- methyl bonded to the carboxylic group at 2.7 ppm

We determined that the product is pure by the absence of signals corresponding to acrylic acid (2 doublets and 1 triplet around 5.5-6.0) and by the absence of a singlet at 2.1 ppm corresponding to methyl groups bonded to the amine in the *N*,*N*-dimethyl(aminoethyl) methacrylate (DMAEMA).

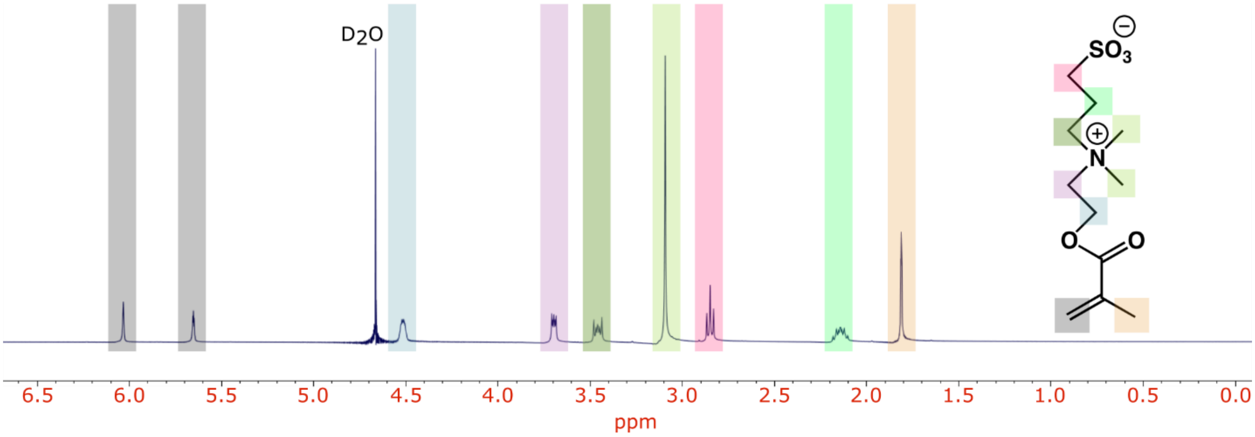

Figure S2: H^1NMR of sulfobetaine methacrylate (SB) in D_2O.

The chemical structure of SB is confirmed by:

- singlet at 3.05 corresponding to the two methyl groups bonded to the amine
- signals at 3.4 and 3.6 corresponding to the two methylene groups
- methacrylate group (5.6 ppm, 6.1 ppm, and 1.7 ppm)
- two methylene groups arising from derivatization with 1,3-propanesultone (2.1 ppm and 2.85 ppm)

We can determine that the product is purified by the absence of a singlet at 2.1 ppm corresponding to the methyl groups bonded to the nitrogen in DMAEMA and the absence of signals corresponding to the 1,3-propanesultone.

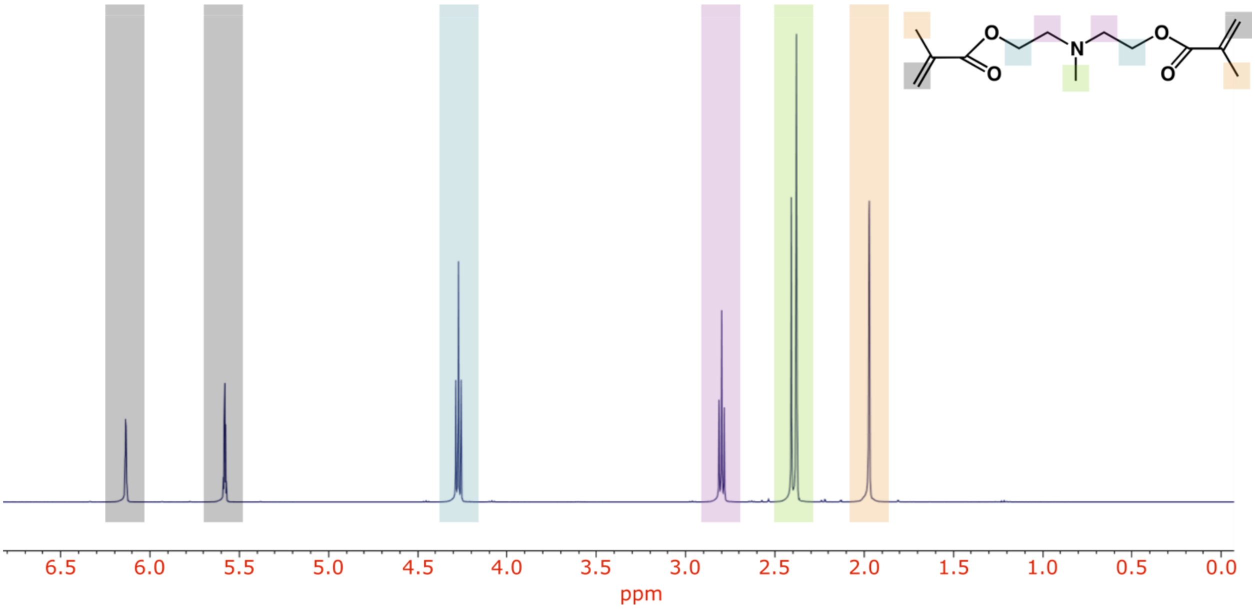

Figure S3: H[1]NMR of *N*-methyldiethanolamine dimethacrylate in $CDCl_3$.

In this NMR, we can see the product of the reaction between methacrylic acid and *N*-methyl diethanolamine. We confirmed:

- successful methacrylation (signals of the methacrylate group at 1.9 ppm, 5.6 ppm, and 6.15 ppm)
- shift of the signal corresponding to the methylene group previously bonded to the alcohol and now to the ester (there is no signal at 3.6 ppm and a signal at 4.3 ppm).
- signals corresponding the methyl and the methylene groups bonded to the amine (2.35 ppm and 2.75 ppm).

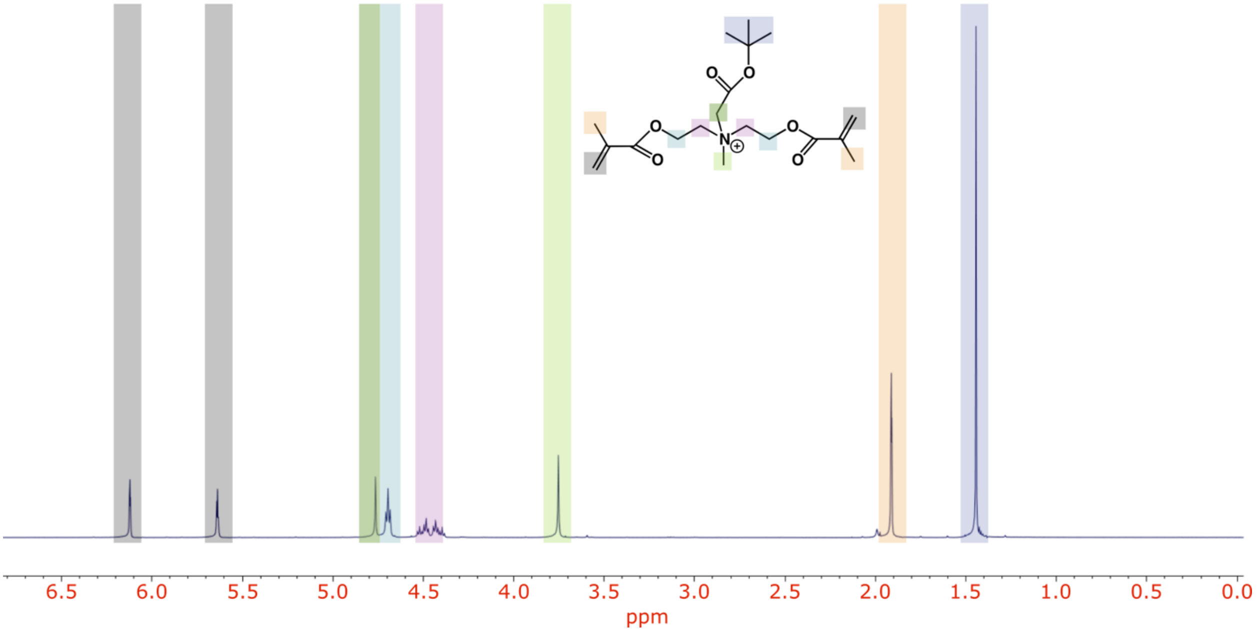

Figure S4: H^1NMR of *N*-Methyl-*N*-di(2-methacryloyloxy-ethyl)-*N*-1-(t-butyloxycarbonylmethyl) ammonium bromide in $CDCl_3$.

In this NMR, we see the product of the quaternization of *N*-methyl diethanolamine methacrylate with tertbutyl ester bromoacetate.

- shift of the signal corresponding to the methyl groups in the amine (from 2.3 ppm in Fig. S3 to 3.7 ppm)
- shift in the two methylene groups bonded to the amine from 2.85 ppm to 4.4 ppm
- shift in the methyl bonded to the ester group from 4.4 ppm to 4.7 ppm
- signal at 1.4 ppm corresponding to the tert-butyl group
- signal at 4.8 ppm corresponding to the methylene group

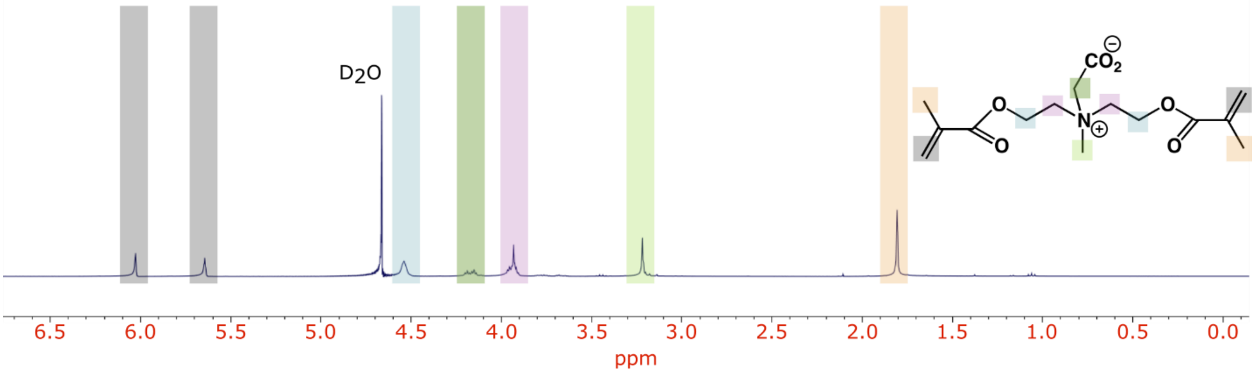

Figure S5: H[1]NMR of carboxybetaine dimethacrylate (CBX) in D_2O.

We see the deprotection of the carboxylic acid by the removal of tert-butyl protecting group with trifluoroacetic acid from **Fig. S4**:

- disappearance of the signal at 1.45 ppm corresponding to the tert-butyl group

Also, the appearance of a negative charge in the carboxylic acid produces different shifts in the surrounding groups:

- shift from 4.6 ppm to 4.2 ppm in the CH_2 group between the quaternary ammonium and the carboxylic acid
- shift from 3.7 ppm to 3.25 ppm in the methyl groups of the quaternary ammonium
- shift from 4.4 ppm to 3.95 ppm of the signal from the CH_2 groups bonded to the quaternary ammonium

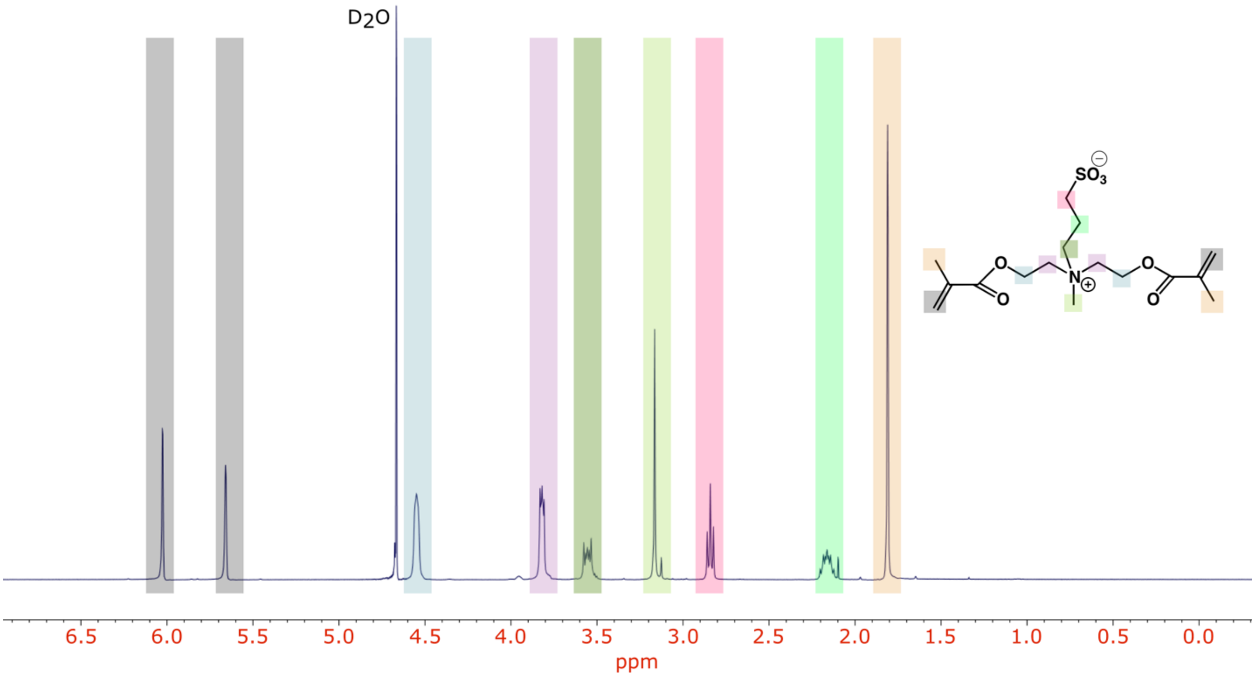

Figure S6: H[1]NMR of sulfobetaine dimethacrylate (SBX) in D_2O.

We can observe the quaternization of the ammonia group (from **Fig. S3**) by the disappearance of the signal at 2.4 ppm and the appearance of a signal at 3.15 ppm, corresponding to the methyl groups bonded to the ammonia.

We can also see the appearance of signals at 2.2 ppm, 2.7 ppm, and 3.55 ppm corresponding to the three CH_2 groups incorporated by the ring-open reaction of 1,3-propanesultone.

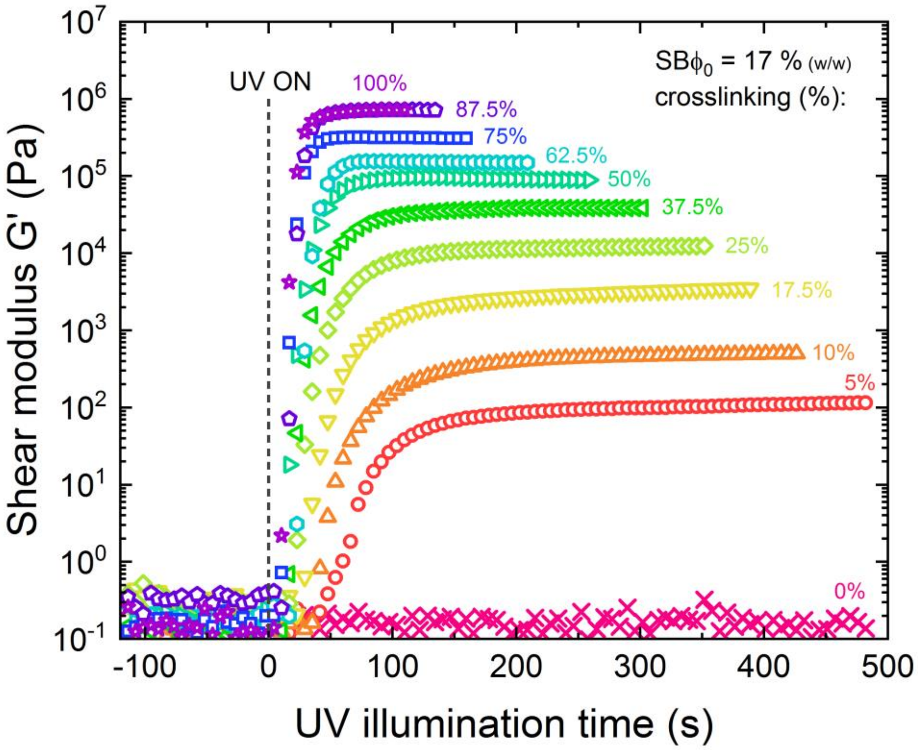

Figure S7: *In situ* photopolymerization of SB/SBX with concentration Φ_0 = 17 % (w/w) and varying crosslinking ratio (adjusted by SBX fraction) indicated in each data series. Mechanical properties and polymerization kinetics of **Fig. 1c** and **Fig. 1d** were calculated from photopolymerization data sets for CB/CBX and SB/SBX at concentrations Φ_0 = 10%, 17%, 40%, and 60% (w/w).

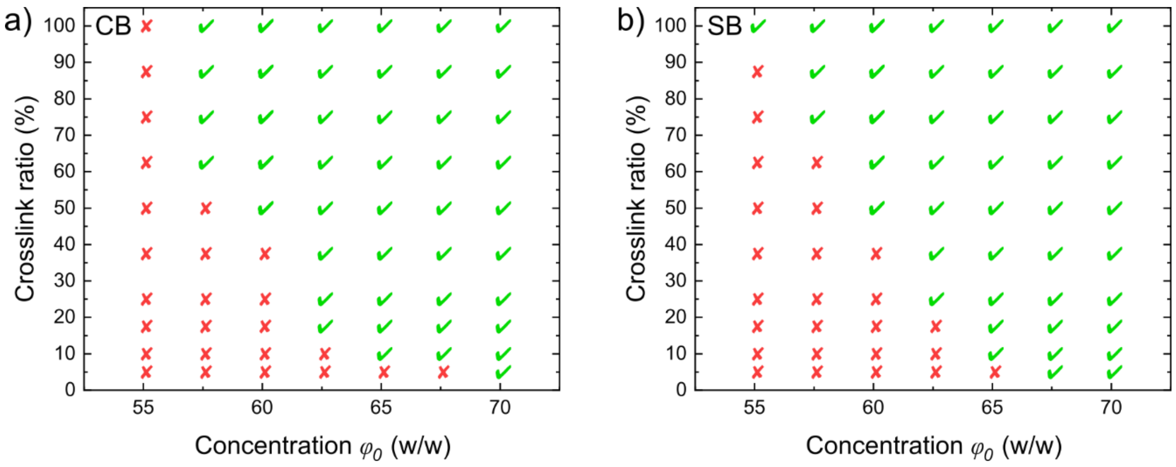

Figure S8: Printing diagram for optimized resolution as a function of concentration and crosslink ratio for **a)** CB-based and **b)** SB-based zwitterionic photoresists, denoting parameters with suboptimal resolution (✗) and optimal resolution with full structural reproducibility (✓).

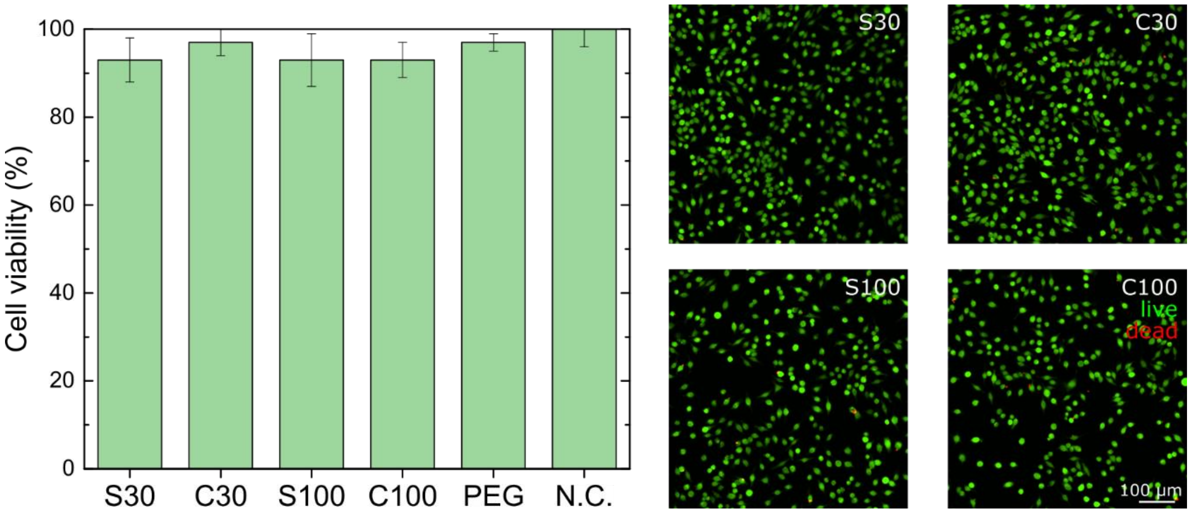

Figure S9: Cell viability of zwitterionic photoresists show that they are non-cytotoxic and biocompatible, with cell viability ≳ 95 %. Live/dead staining shows an overwhelming majority of live cells (J774A.1 murine macrophages) after co-culture with the photoresists. WST-8 viability counts agreed with live/dead counts, confirming the biocompatibility of the tested materials.

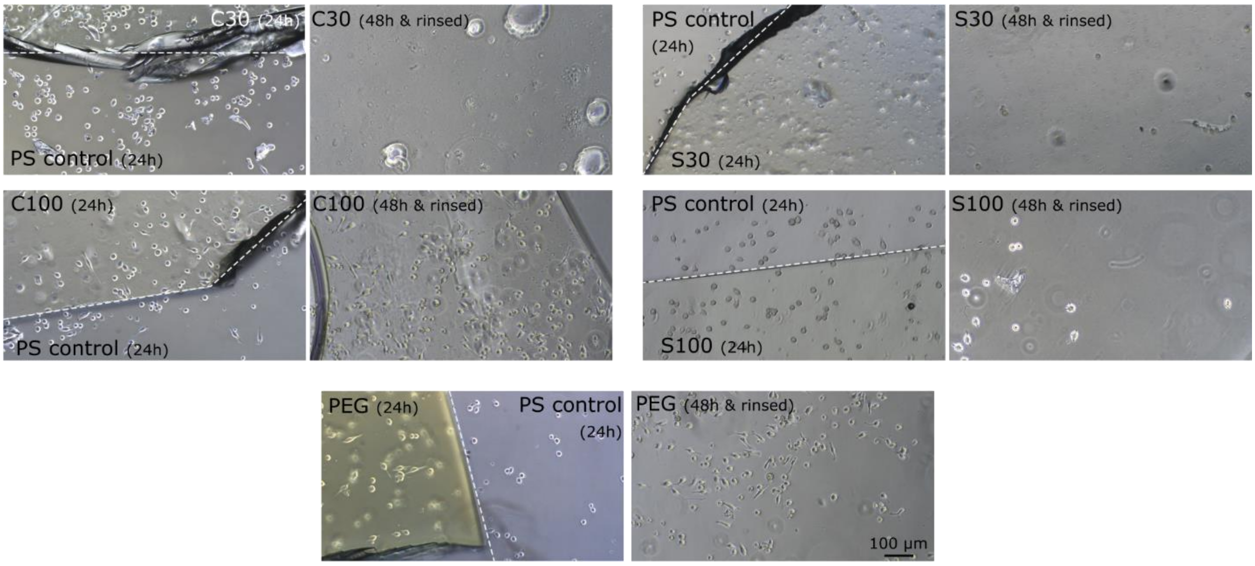

Fig. S10: Cell adhesion to zwitterionic photoresists. J774A.1 murine macrophages were cultured on photopolymerized C30, S30, C100, S100, and PEGDA hydrogels in polystyrene (PS) plates for 24 hours (first image was taken). After 48 hours the photopolymerized films were rinsed and transferred to new plates to observe cell adhesion. C30, S30, and S100 have no cells after rinsing, indicating very low cell adhesion to the material. However, C100 exhibits some cell adhesion after 48 hours, possibly due to batch synthesis impurities. PEG exhibits high cell adhesion, observable in the elongated morphology of the attached cells.

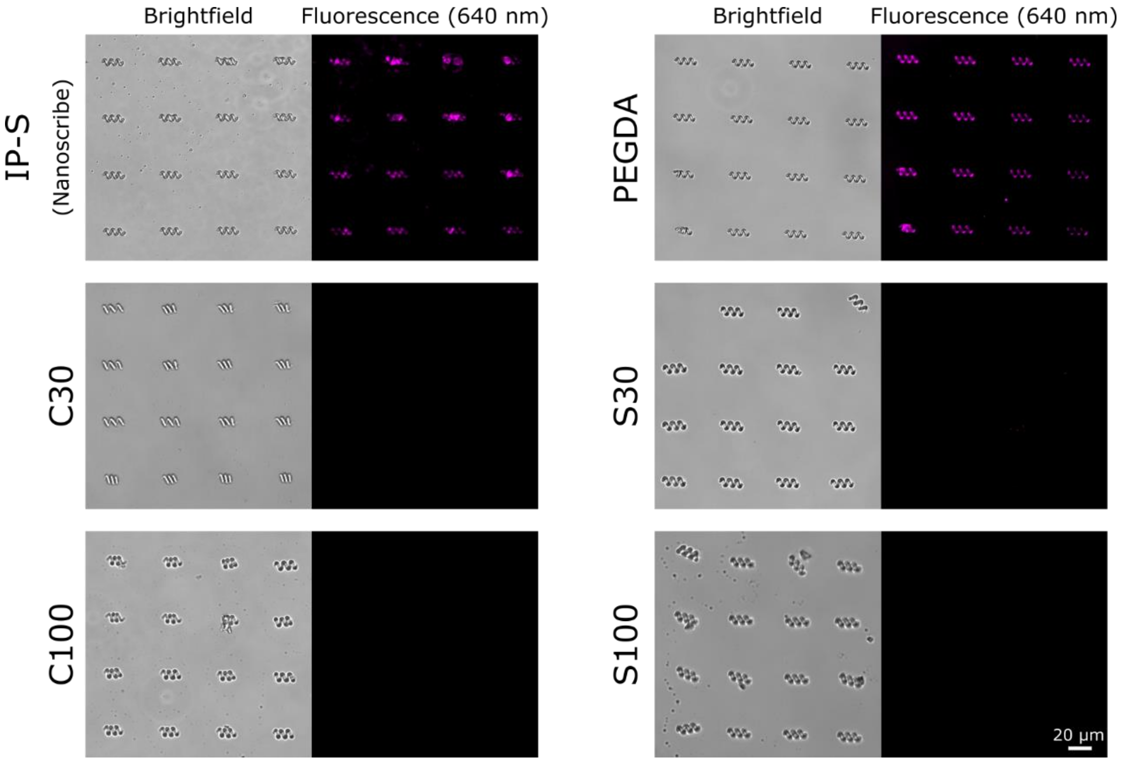

Figure S11: Protein adsorption on zwitterionic microrobots. To avoid conflict with any possible background signal from photoinitiators, we tested the adsorption of fluorescent Cy5-modified BSA (no fluorescence overlap). Commercially available photoresists (IP-S, Nanoscribe GmbH) and PEG-based photoresists showed protein adsorbed on their surface, evident from fluorescence intensity in the images. However, none of the zwitterionic microrobots showed any fluorescence intensity, indicating that the protein was not adsorbed on their surface due to their anti-biofouling properties.

Note S1

The immune system is extremely complex and exerts multiple functions in order to maintain homeostasis. The recognition of both foreign (pathogens, foreign bodies) and host (cancerous cells, cell debris) threats and their removal is a key component of host immunity[1]. In order to perform all these activities, it comprises a wide variety of immune cells with different behaviors and functions. However, macrophages are the most relevant immune cells regarding foreign synthetic biomaterials, such as nanoparticles, microparticles, or in this case microrobots, as we discuss below.
The immune system is composed of an *innate* and an *adaptive* component, as extensively described in immunology literature[1]. The innate immune system provides the first line of defense with fast *non-specific* mechanisms and broad reactivity, while the adaptive immune system has a high degree of *specificity,* but it is slow to develop and requires an antigenic challenge. The innate immune system includes phagocytic cells (such as monocytes, macrophages, and neutrophils) that internalize and eliminate the threat, while the major agents of the adaptive immune system are lymphocytes that can produce antibodies.
The mononuclear phagocyte system (MPS), which belongs to the innate immune system (non-specific), is responsible for the recognition and clearance of the foreign materials from the body[2–5], and is composed majorly of monocytes and macrophages[6]. Monocytes circulate in the bloodstream for about 8 hours where they can phagocyte hazardous particles, and then migrate to tissues to differentiate into specific tissue-resident macrophages or dendritic cells[7]. Macrophages, which are present in almost all body tissues and especially in lungs, liver and spleen, are activated when they detect a material as a foreign body, mainly through the identification of adsorbed proteins on the material surface[8,9], and then proceed to eliminate the foreign material by phagocytosis. Macrophages are also capable of ingesting and digesting exogenous microorganisms (bacterial cells or fungi components) and endogenous matter (such as dead cells or cellular debris), however, this discussion focuses on exogenous materials, as this is the case for synthetic microrobots.
Monocytes and macrophages also perform two additional functions when they recognize and phagocytose a foreign body: the secretion of cytokines (regulators of the immune system) and the presentation of antigens (small peptides of the phagocytosed material). These lymphocytes will then mount a highly specific immune response against that specific antigen they were presented. This step is crucial for the adaptive immune response since lymphocytes are nonphagocytic cells and require the antigen presentation to secrete specific antibodies and perform cytotoxic functions. They have an immunogenic memory that allows them to generate antibodies against the same antigen time after the first exposure (to avoid second infections).

Therefore, although lymphocytes play a very important role as indicated by a reviewer, they function differently than macrophages, since they are nonphagocytic and their activation is

antigen-driven. It is the function of the MPS to phagocytose and eliminate foreign bodies and to present antigens that will activate the lymphocytes. This is the main reason why there have been many efforts to characterize and modulate the interaction between macrophages and biomaterials[3,8,10–17], and also why we have focused on macrophages to demonstrate the immune evasive properties of our zwitterionic stealth microrobots. However, to provide a more comprehensive analysis of the stealth properties and to validate the broader non-immunogenicity of our microrobots, we have expanded our study to different immune cells, including *monocytes* (which are part of the mononuclear phagocyte system) and *splenocytes* (which consist of a diverse collection of immune cells present in the spleen, including include T-lymphocytes, B-lymphocytes, dendritic cells, and macrophages).

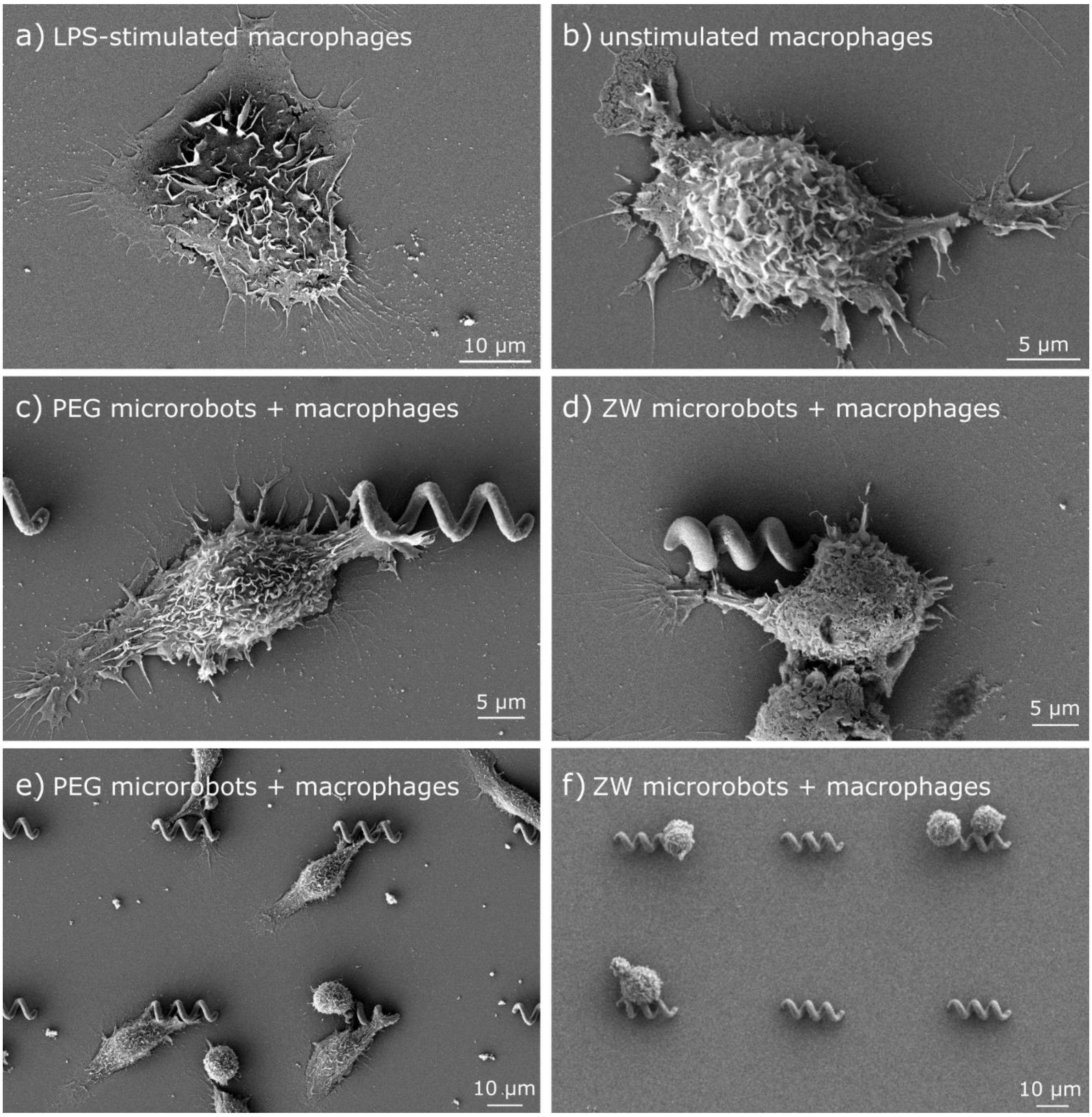

Figure S12: SEM images of: **a)** LPS-stimulated macrophage, **b)** unstimulated macrophage, **c)** macrophage interacting with non-stealth PEG microrobot, **d)** macrophage interacting with stealth S30 microrobot, **e)** macrophages interacting with PEG microrobot arrays, and **f)** macrophages interacting with stealth microrobot arrays. The images were taken at the early stages of cell inspection (when macrophages come in contact with the robots but before they are phagocytosed). When inspecting PEG microrobots, macrophages show more aggressive behavior. This is indicated by: a) migration towards the microrobot resulting in elongated morphology, b) extension of filopodia and lamellipodia towards the microrobot, and c) presentation of crisp dorsal ruffles on the cell surface[18,19]. These features on the cell surface (optimized for probing and initiating phagocytosis), are observed in activated macrophages[20,21] such as lipopolysaccharide (LPS)-stimulated macrophages. In contrast, macrophages inspecting zwitterionic microrobots present smoother cell surfaces, with few or none filopodia trying to engulf the robot.

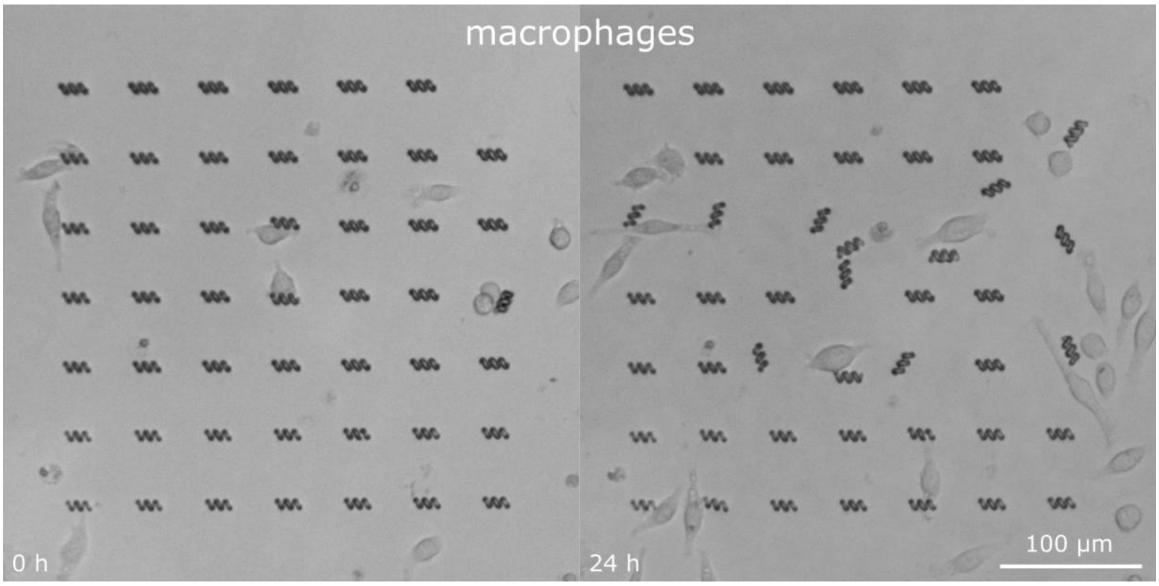

Figure S13: S30 microrobot arrays co-cultured with macrophages for 24 hours. We observe that macrophages exhaustively inspect the robots by probing them and moving them around (also visible in **Movie S2**). However, the microrobots are released after inspection as they are not detected as a threat, thus avoiding phagocytosis. After 24 hours, many robots have been displaced by the cells, but none of them have been phagocytosed.

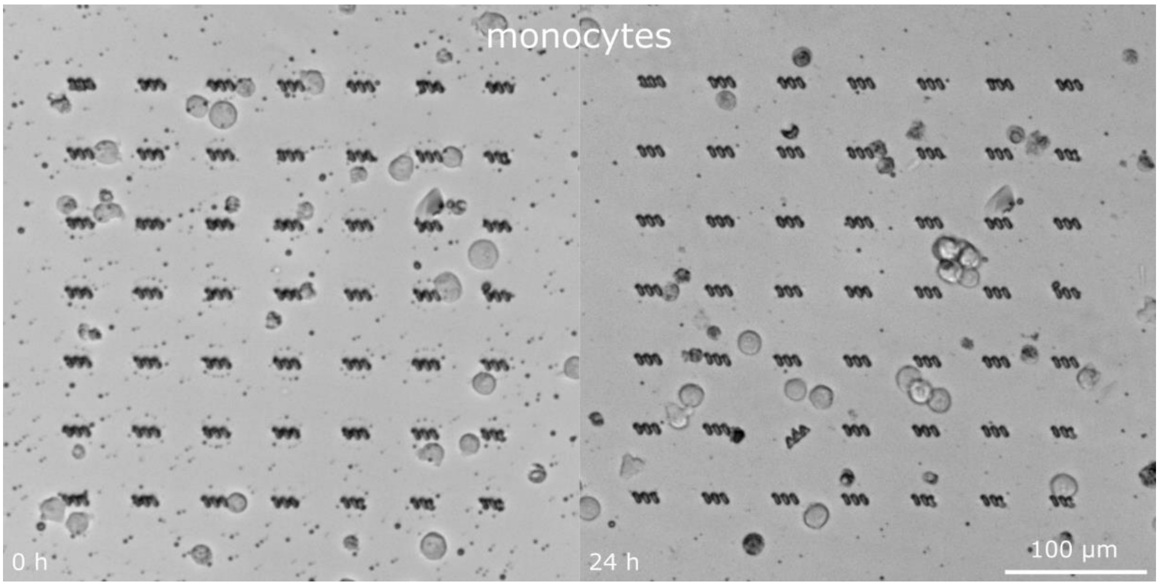

Figure S14: S30 microrobot arrays co-cultured with monocytes for 24 hours. We observe that macrophages exhaustively inspect the robots by probing them and moving them around (also visible in **Movie S2**). However, the microrobots are released after inspection as they are not detected as a threat, thus avoiding phagocytosis. After 24 hours, robots have been displaced by the cells, but none of them have been phagocytosed. Furthermore, monocytes clear the surrounding debris (visible at 0 h) but do not phagocytose the robots, which further validates their stealth properties.

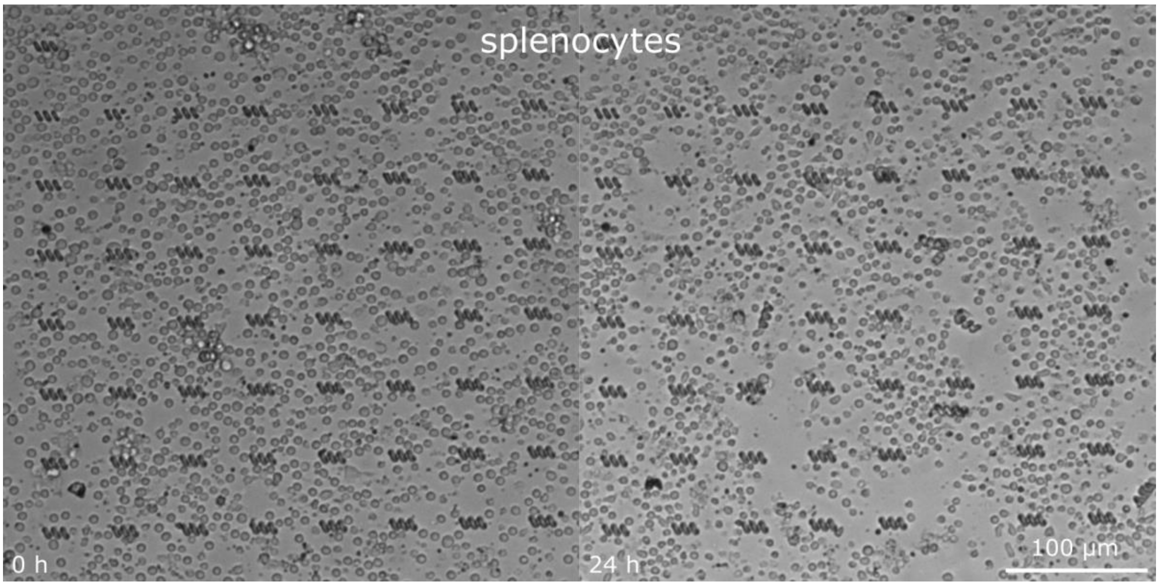

Figure S15: S30 microrobot arrays co-cultured with splenocytes for 24 hours. During this period, phagocytosis was not observed even at high cell densities and after repeated inspection of the microrobots by multiple cells. In some cases (more appreciable in **Movie S2**), microrobots are inspected by the cells, moved around, and then released without phagocytosis, which clearly demonstrates that they are not recognized as a foreign threat.

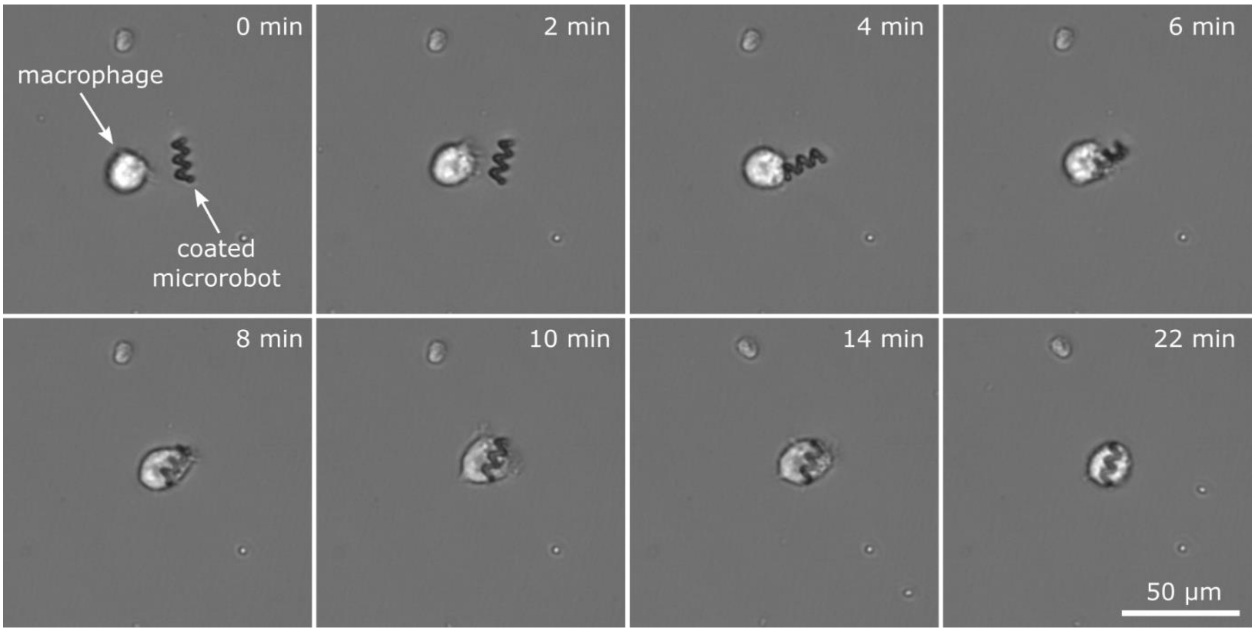

Figure S16: Interaction between a macrophage and a red blood cell (RBC) membrane-coated PEG-based microrobot. The microrobot is immediately phagocytosed as soon as it comes in contact with the macrophage. The microrobot is immediately recognized by the macrophage as soon as they come in contact, and the robot is phagocytosed shortly after. This is also observed in other microrobot systems with specialized coatings such as PEG-coated, where similar microrobots are also phagocytosed[13].

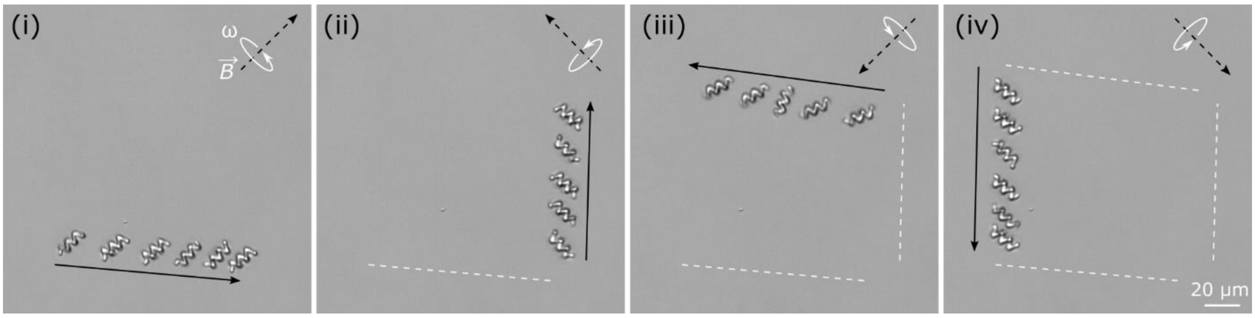

Figure S17: Microrobot rolling locomotion along a programmed square trajectory. For each orthogonal change of direction, the magnetic field rotation axis was turned 90°.

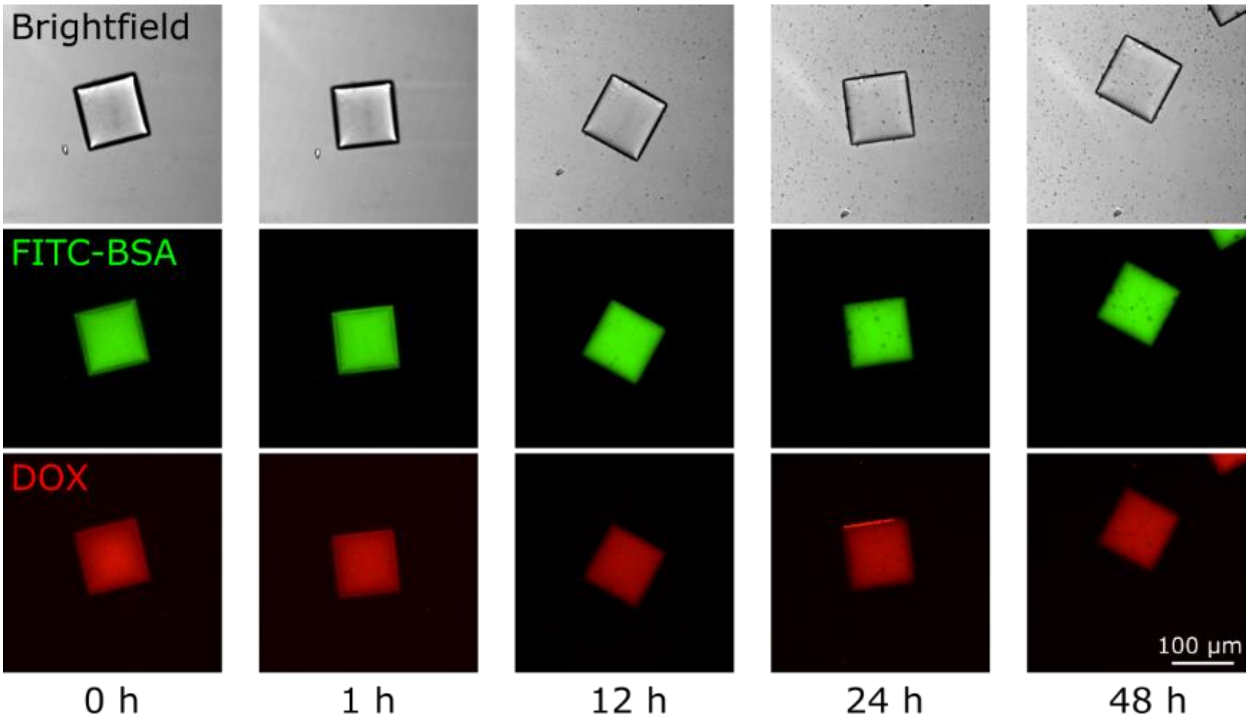

Figure S18: Stability of simultaneously encapsulated biomolecules (FITC-BSA protein, 66 kDa, and doxorubicin, 544 Da) in 3D-printed C30 hydrogels. Constant fluorescence intensity over a 48 hours period indicates that both biomolecules are not leaking out of the hydrogel network.

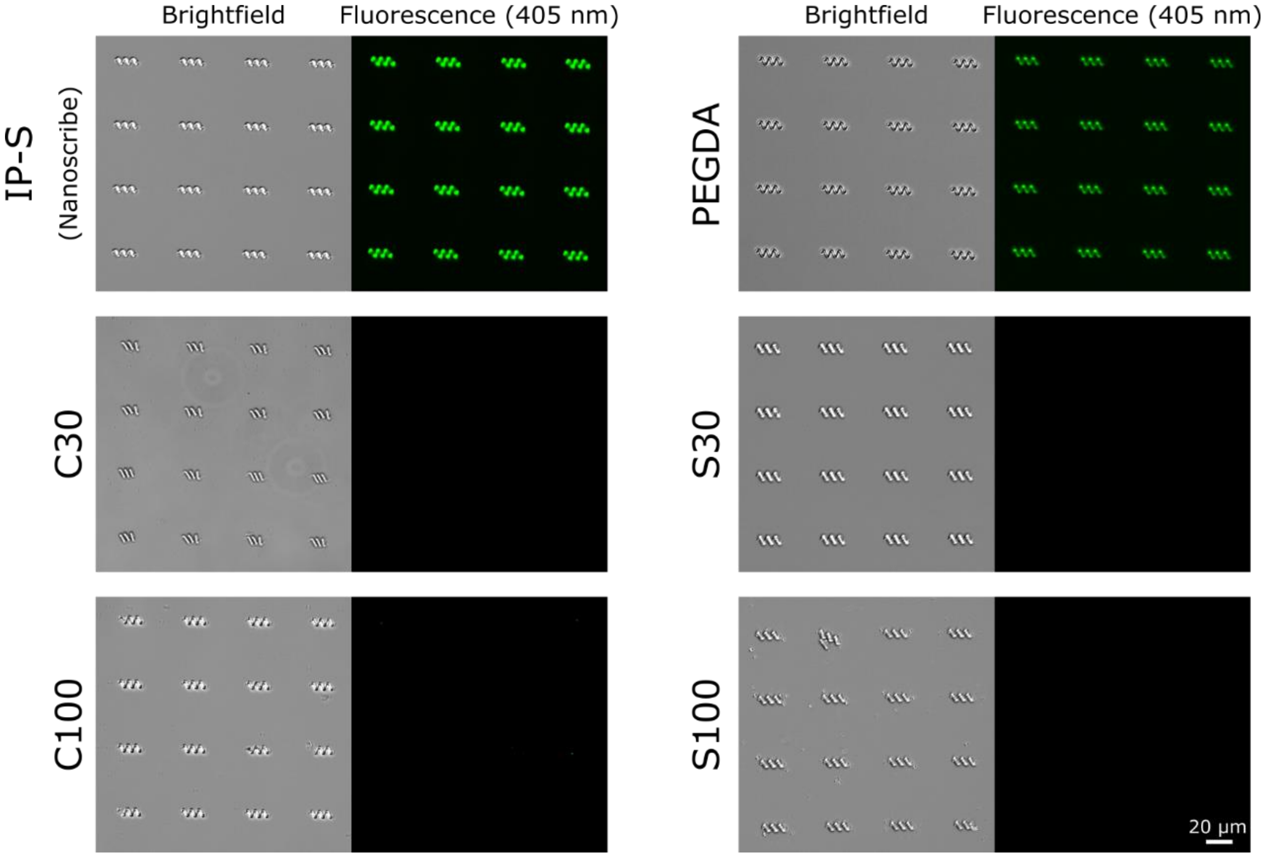

Figure S19: Residual photoinitiator in 3D-printed microrobots. Commercially available photoresists (IP-S, Nanoscribe GmbH) and PEG-based synthetic photoresists (polyethylene glycol diacrylate [PEGDA]) exhibit fluorescence from residual photoinitiator in their structure, which complicates fluorescence microscopy characterization due to unwanted background. Zwitterionic photoresists do not exhibit residual fluorescence and therefore are compatible with diverse fluorescence microscopy analyses of fluorescent biomolecules and fluorophores interacting with microrobots.

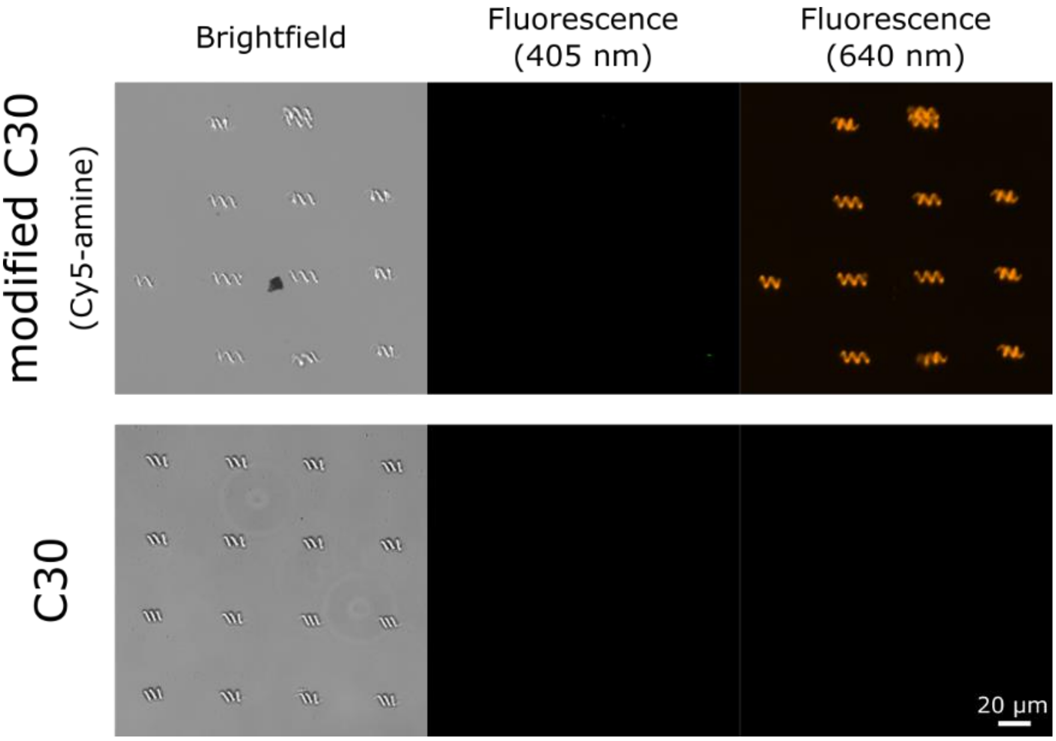

Figure S20: Surface functionalization of C30 microrobots with Cy5-amine fluorophore (from Lumiprobe) *via* EDC/NHS chemistry (carboxylic group from CB with amine group from fluorophore). Non-modified C30 microrobots do not exhibit any fluorescence. Modified C30 microrobots exhibit fluorescence only at 640 nm (corresponding to Cy5).

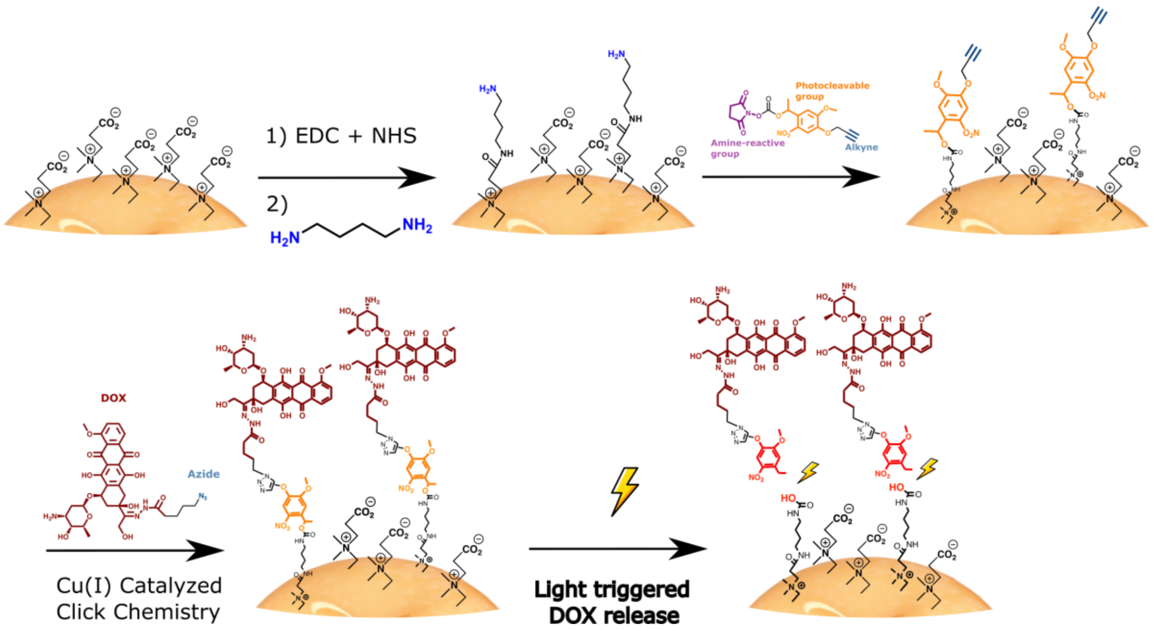

Figure S21: Surface functionalization of C30 microrobots with doxorubicin through a photocleavable linker for light-triggered drug delivery. We used a diamine to bind the linker to the CB surface through its carboxylic group. Butyldiamine was selected due to a low MW and solubility in water. Once we introduced free amines on the surface of the microrobots using EDC/NHS chemistry, the linker was bonded to the free amine also using the NHS end. Next, copper (I) catalyzed azide-alkyne click chemistry was performed to bond azide-modified doxorubicin with the alkyne end of the photocleavable linker. After bonding doxorubicin to the surface of C30 microrobots through a photocleavable linker, we demonstrated light-triggered doxorubicin release by UV illumination (**Figure S22**).

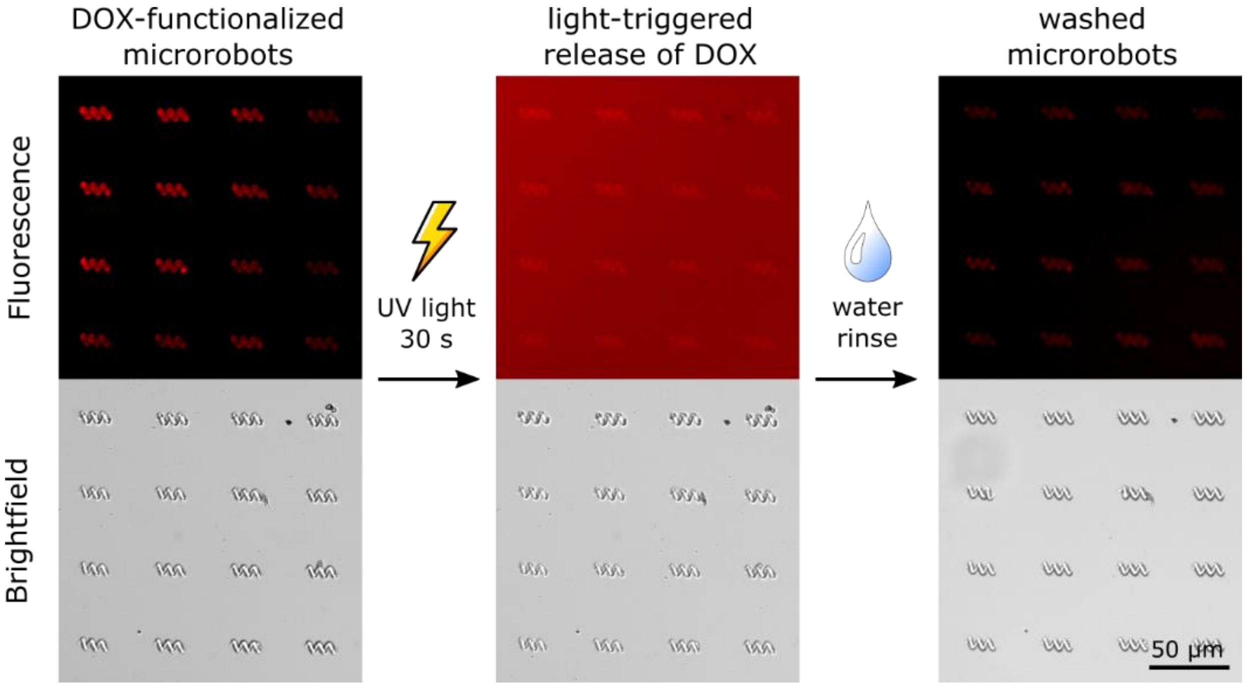

Figure S22: Light-triggered release of doxorubicin (DOX) in functionalized C30 microrobots. Initially bound to the microrobot surface, fluorescent DOX molecules are released to the media after UV light (30 seconds, 55 mW/cm^2) exposure. After a water rinse, washed microrobots exhibit decreased fluorescence intensity due to the released DOX molecules.

Supplementary Movies

Movie S1. 3D-microprinting of zwitterionic hydrogels

Two-photon polymerization of zwitterionic photoresists into 3D microstructures. Examples show the printing of a 3D helical microrobot (20 μm in length) and the MPI's Minerva (50 μm in diameter) from CB30 photoresist.

Movie S2. Stealth microrobots for macrophage evasion

Interactions between the zwitterionic and PEG microrobots and macrophages. Control microrobots (PEG-based) are detected after a short inspection and captured by activated macrophages. Zwitterionic microrobots remain undetected after multiple and exhaustive inspections and are released, evading capture by different immune cells (macrophages, monocytes, and splenocytes).

Movie S3. Zwitterionic microrobot actuation & locomotion

Magnetic actuation of zwitterionic microrobots (functionalized with magnetic nanoparticles) *via* rotating magnetic fields. At low frequencies (ω = 3 Hz) the robot exhibits rolling locomotion. At higher frequencies (ω = 13 Hz) the robot exhibits corkscrew swimming locomotion.

Supplementary References

[1] T. J. Kindt, R. A. Goldsby, B. A. Osborne, J. Kuby, *Kuby Immunology*, Macmillan, **2007**.
[2] R. L. Juliano, *Adv. Drug Deliv. Rev.* **1988**, *2*, 31.
[3] Y. Tabata, Y. Ikada, in *New Polym. Mater.*, Springer-Verlag, Berlin/Heidelberg, **1990**, pp. 107–141.
[4] S. Nie, *Nanomedicine* **2010**, *5*, 523.
[5] E. Blanco, H. Shen, M. Ferrari, *Nat. Biotechnol.* **2015**, *33*, 941.
[6] D. A. Hume, *Curr. Opin. Immunol.* **2006**, *18*, 49.
[7] I. Carr, *macrophage. A Rev. Ultrastruct. Funct.* **1973**.
[8] J. E. Rayahin, R. A. Gemeinhart, *Macrophages*, Springer International Publishing, Cham, **2017**.
[9] Z. J. Deng, M. Liang, M. Monteiro, I. Toth, R. F. Minchin, *Nat. Nanotechnol.* **2011**, *6*, 39.
[10] W. J. Kao, *Biomaterials* **1999**, *20*, 2213.
[11] R. Klopfleisch, *Acta Biomater.* **2016**, *43*, 3.
[12] J. M. Anderson, K. M. Miller, in *Biomater. Silver Jubil. Compend.*, Elsevier, **1984**, pp. 21–26.
[13] I. C. Yasa, H. Ceylan, U. Bozuyuk, A.-M. Wild, M. Sitti, *Sci. Robot.* **2020**, *5*, eaaz3867.
[14] P. Pacheco, D. White, T. Sulchek, *PLoS One* **2013**, *8*, e60989.
[15] M. E. Ogle, C. E. Segar, S. Sridhar, E. A. Botchwey, *Exp. Biol. Med.* **2016**, *241*, 1084.
[16] O. Lunov, T. Syrovets, C. Loos, J. Beil, M. Delacher, K. Tron, G. U. Nienhaus, A. Musyanovych, V. Mailänder, K. Landfester, T. Simmet, *ACS Nano* **2011**, *5*, 1657.
[17] J. M. Anderson, A. Rodriguez, D. T. Chang, *Semin. Immunol.* **2008**, *20*, 86.
[18] J. L. Stow, N. D. Condon, *Clin. Transl. Immunol.* **2016**, *5*, e71.
[19] N. D. Condon, J. M. Heddleston, T.-L. Chew, L. Luo, P. S. McPherson, M. S. Ioannou, L. Hodgson, J. L. Stow, A. A. Wall, *J. Cell Biol.* **2018**, *217*, 3873.
[20] D. V Krysko, G. Denecker, N. Festjens, S. Gabriels, E. Parthoens, K. D'Herde, P. Vandenabeele, *Cell Death Differ.* **2006**, *13*, 2011.
[21] P. C. Patel, R. E. Harrison, *Mol. Biol. Cell* **2008**, *19*, 4628.
